# The TLR4 adaptor TRAM controls the phagocytosis of Gram-negative bacteria by interacting with the Rab11-family interacting protein 2

**DOI:** 10.1371/journal.ppat.1007684

**Published:** 2019-03-18

**Authors:** Astrid Skjesol, Mariia Yurchenko, Korbinian Bösl, Caroline Gravastrand, Kaja Elisabeth Nilsen, Lene Melsæther Grøvdal, Federica Agliano, Francesco Patane, Germana Lentini, Hera Kim, Giuseppe Teti, Aditya Kumar Sharma, Richard K. Kandasamy, Bjørnar Sporsheim, Kristian K. Starheim, Douglas T. Golenbock, Harald Stenmark, Mary McCaffrey, Terje Espevik, Harald Husebye

**Affiliations:** 1 Centre of Molecular Inflammation Research, Department of Clinical and Molecular Medicine, Norwegian University of Science and Technology, Trondheim, Norway; 2 Department of Clinical and Experimental Medicine, University of Messina, Messina, Italy; 3 Program in Innate Immunity, Division of Infectious Diseases and Immunology, Department of Medicine, University of Massachusetts Medical School, Worcester, MA, United States of America; 4 Centre for Cancer Cell Reprogramming, Faculty of Medicine, University of Oslo, Oslo, Norway; 5 Department for Molecular Cell Biology, Institute for Cancer Research, Oslo University Hospital, Oslo Norway; 6 Molecular Cell Biology Laboratory, Biochemistry Department, Biosciences Institute, University College Cork, Cork, Ireland; 7 The Central Norway Regional Health Authority, St. Olavs Hospital HF, Trondheim, Norway; University of Toronto, CANADA

## Abstract

Phagocytosis is a complex process that eliminates microbes and is performed by specialised cells such as macrophages. Toll-like receptor 4 (TLR4) is expressed on the surface of macrophages and recognizes Gram-negative bacteria. Moreover, TLR4 has been suggested to play a role in the phagocytosis of Gram-negative bacteria, but the mechanisms remain unclear. Here we have used primary human macrophages and engineered THP-1 monocytes to show that the TLR4 sorting adapter, TRAM, is instrumental for phagocytosis of *Escherichia coli* as well as *Staphylococcus aureus*. We find that TRAM forms a complex with Rab11 family interacting protein 2 (FIP2) that is recruited to the phagocytic cups of *E*. *coli*. This promotes activation of the actin-regulatory GTPases Rac1 and Cdc42. Our results show that FIP2 guided TRAM recruitment orchestrates actin remodelling and IRF3 activation, two events that are both required for phagocytosis of Gram-negative bacteria.

## Author summary

The Gram-negative bacteria *E*. *coli* is the most common cause of severe human pathological conditions like sepsis. Sepsis is a clinical syndrome defined by pathological changes due to systemic inflammation, resulting in paralysis of adaptive T-cell immunity with IFN-β as a critical factor. TLR4 is a key sensing receptor of lipopolysaccharide on Gram-negative bacteria. Inflammatory signalling by TLR4 is initiated by the use of alternative pair of TIR-adapters, MAL-MyD88 or TRAM-TRIF. MAL-MyD88 signaling occurs mainly from the plasma membrane giving pro-inflammatory cytokines like TNF, while TRAM-TRIF signaling occurs from vacuoles like endosomes and phagosomes to give type I interferons like IFN-β. It has previously been shown that TLR4 can control phagocytosis and phagosomal maturation through MAL-MyD88 in mice, however, these data have been disputed and published before the role of TRAM was defined in the induction of IFN-β. A role for TRAM or TRIF in phagocytosis has not previously been reported. Here we describe a novel mechanism where TRAM and its binding partner Rab11-FIP2 control phagocytosis of *E*. *coli* and regulate IRF3 dependent production of IFN-β. The significance of these results is that we define Rab11-FIP2 as a potential target for modulation of TLR4-dependent signalling in different pathological states.

## Introduction

Phagocytosis is a complex and versatile process that eliminates pathogens and is performed by specialized cells such as macrophages [1]. Phagocytosis requires cell surface receptors recognizing the pathogen [2] and Rho GTPases controlling local actin dynamics that drive engulfment [2–5]. Toll-like receptor 4 (TLR4) recognizes lipopolysaccharide (LPS) present on Gram-negative bacteria [6], and data from mouse macrophages show that TLR4 is required for the phagocytosis of *E*. *coli* [7, 8]. Moreover, LPS-stimulated phagocytosis of *E*. *coli* occurs through actin polymerization controlled by Rho GTPases, Rac1 and Cdc42, although the mechanisms are unclear [9].

In human macrophages, Rab11 is recruited to *E*. *coli* phagosomes and controls TLR4-mediated induction of interferon-β (IFN-β) [10]. Like all GTPases, Rab11 acts as a molecular switch alternating between active (GTP-bound)- and inactive (GDP-bound) forms [11]. In the active state Rab11 binds effector proteins such as the Rab11-family interacting proteins (FIPs), allowing Rab11 to recruit cellular motor proteins [12]. FIP2 regulates intracellular transport within the recycling system and links Rab11 to actin motor proteins, like Myosin5B, to coordinate vesicle trafficking [13–16]. FIP2 also controls EGFR-mediated endocytosis [14] and EGFR-mediated internalization of *Chlamydia pneumoniae* [17].

Activation of TLR4 results in two different signalling pathways depending on cellular location and the recruited pair of Toll/interleukin-1 receptor (TIR) domain-adaptors [10, 18, 19]. At the plasma membrane, TLR4 binds MyD88-adaptor-like (Mal) and MyD88 to drive NF-κB activation and subsequent production of proinflammatory cytokines, such as TNF. From endosomes TLR4 binds TRIF-related adaptor molecule (TRAM) and TIR-domain-containing adapter-inducing interferon-β (TRIF) to initiate the production type I interferons, like IFN-β, through activation of the Interferon regulatory factor 3 (IRF3). A direct action of TRAM or TRIF in phagocytosis has not been established. Here we provide evidence that TRAM is a critical regulator of *E*. *coli* phagocytosis by a mechanism dependent on FIP2. We find that TRAM interacts with FIP2 to drive actin filament formation at forming phagosomes through activation of Rac1 and Cdc42. As a consequence, the TRAM-FIP2 complex is instrumental in controlling both phagocytosis and TLR4-mediated TRAM-TRIF signalling from *E*. *coli* phagosomes.

## Results

### TRAM and FIP2 are recruited to F-actin positive membrane foci at forming *E*. *coli* phagosomes

We have previously shown that *E*. *coli*-induced IFN-β mRNA expression is controlled by Rab11a and dependent on F-actin-polymerization [10]. Rab11 uses the FIPs as effector molecules to control endocytosis and endosomal sorting [20]. To identify if a single FIP could be involved in the regulation of *E*. *coli*-stimulated IFN-β mRNA expression, PMA differentiated THP-1 cells were silenced for FIP1, FIP2, FIP3, FIP4 and FIP5, and the effect on *E*. *coli*-stimulated IFN-β and TNF mRNA induction was analysed. Of all the FIPs investigated, FIP2 silening had a selective effect on the induction of IFN-β mRNA (S1 Fig). FIP5 silencing reduced IFN-β mRNA expression to a similar extent as FIP2, however, no selectivity was observed as TNF expression also was reduced under this condition (S1 Fig). As shown, reducing FIP2 mRNA expression impaired IFN-β for both *E*. *coli* and LPS stimulations, however, the FIP5 mRNA expression was not affected under this condition of FIP2 silencing.

**Fig 1:**
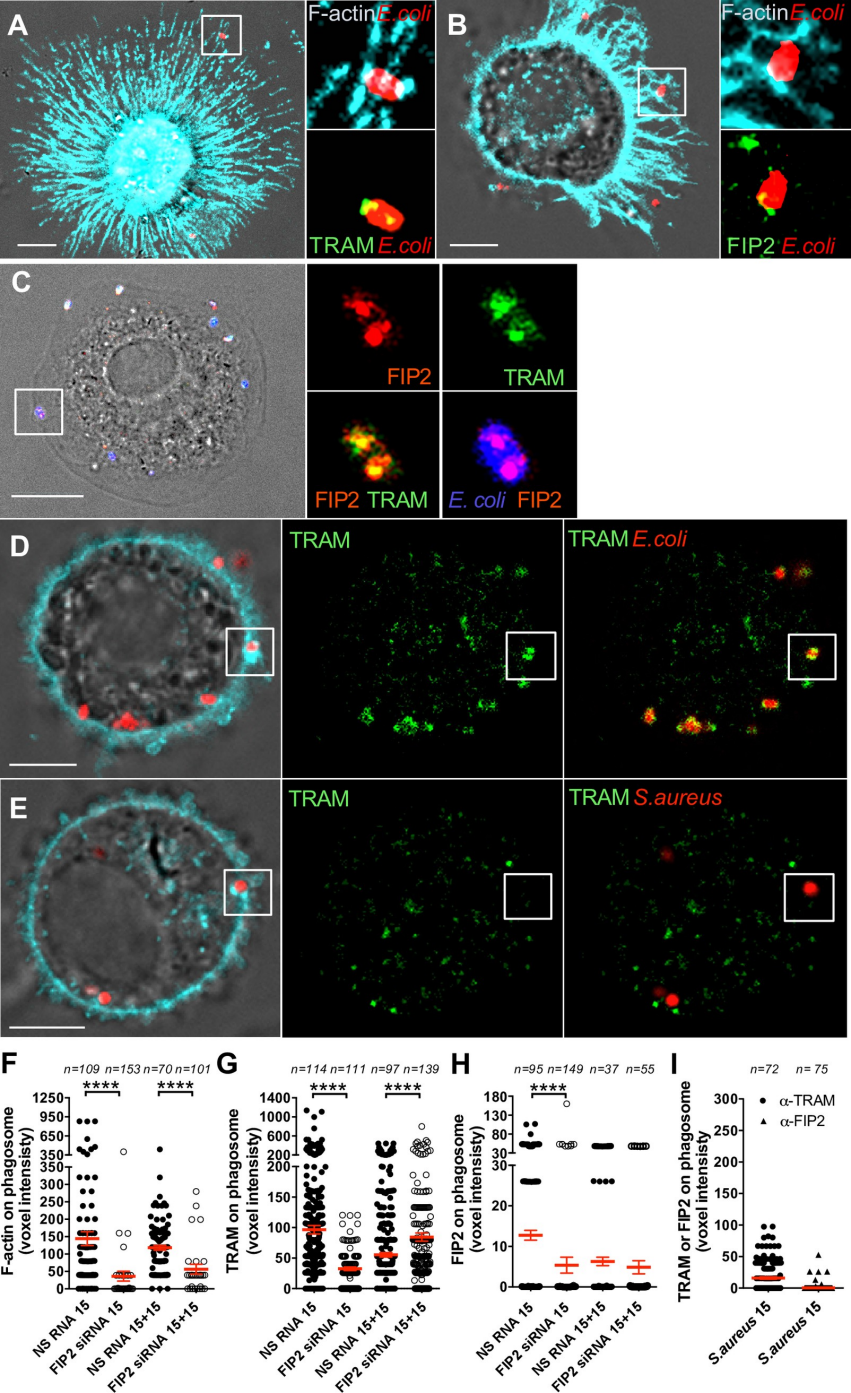
TRAM and FIP2 are recruited to F-actin positive membrane foci during *E*. *coli* phagocytosis. *E*. *coli or S*. *aureus* bioparticles were added to human primary macrophages (Mϕ) as indicated and stained for F-actin using phalloidin (cyan), and immunostained for TRAM or FIP2 (green). (**A)** TRAM and F-actin co-localization at *E*. *coli* binding site 15 min after stimulation. (**B)** FIP2 and F-actin co-localization at *E*. *coli* binding site 15 min after stimulation. (**C)** TRAM and FIP2 co-localization at *E*. *coli* binding sites 15 min after stimulation. (**D)** TRAM and F-actin co-localization at forming *E*. *coli* phagosomes 15+15 min after stimulation. (**E)** TRAM is not detected on *S*. *aureus* phagosomes. (**F)** F-actin-, (**G**) TRAM- and (**H)** FIP2-levles on *E*. *coli* phagosomes in Mϕ treated with NS RNA or FIP2 siRNA and stimulated for 15 and 15+15 min. **(I)** Levels of TRAM and FIP2 on *S*. *aureus* phagosomes 15 min after stimulation. (F-I) Median voxel intensities of TRAM and FIP2 on phagosomes were obtained by 3-D confocal microscopy and quantified using the IMARIS imaging software. n = number of cells monitored per condition. One-way ANOVA Kruskal-Wallis test with adj. p values, **** (p < 0.0001). Red bars = mean ± SEM from three representative human donors. Scale bars = 5 μm.

Since FIP2 was involved in the control of *E*. *coli*-induced IFN-β mRNA induction, we next examined the role of FIP2 in F-actin and TRAM dynamics during *E*. *coli* phagocytosis in primary human macrophages. Surprisingly, TRAM and F-actin co-localized at the *E*. *coli* binding site on plasma membrane protrusions (Fig 1A), and a similar phenotype was observed for FIP2 (Fig 1B). These data suggest that TRAM and FIP2 are rapidly recruited to F-actin foci positive phagocytic cups containing *E*. *coli*. Indeed, TRAM and FIP2 co-localized in distinct spots on developing *E*. *coli* phagosomes 15 min after stimulation (Fig 1C). After a 15 min chase (15+15), where *E*. *coli* was removed by washing and the cells further incubated for 15 min, the amounts of FIP2 showed a marked decay while the amounts of TRAM showed a slight increase at the *E*. *coli* phagosomes (Fig 1G and 1H). Pronounced accumulation of F-actin on the plasma membrane was largely observed at initial phases of uptake (Fig 1D and 1E). In contrast, similarly stimulated *Staphylococcus aureus* macrophages did not show accumulation of TRAM or FIP2 on phagosomes, despite pronounced accumulation of F-actin (Fig 1E and 1I). The observation that both TRAM and FIP2 were recruited to F-actin foci surrounding *E*. *coli* during phagocytosis, led us to investigate if FIP2 silencing could alter TRAM recruitment.

FIP2-silenced macrophages were stimulated by *E*. *coli* as above, before 3-D imaging by confocal microscopy. The FIP2-silenced macrophages showed a marked reduction in both F-actin and TRAM recruitment to *E*. *coli* phagosomes (Fig 1F and 1G). The lack of FIP2 recruitment to phagosomes in FIP2-silenced cells confirmed the specificity of the FIP2 antibody and efficient silencing (Fig 1H).

### Super resolution microscopy reveals that TRAM and TLR4 are differentially organized at the phagosome

**Fig 2:**
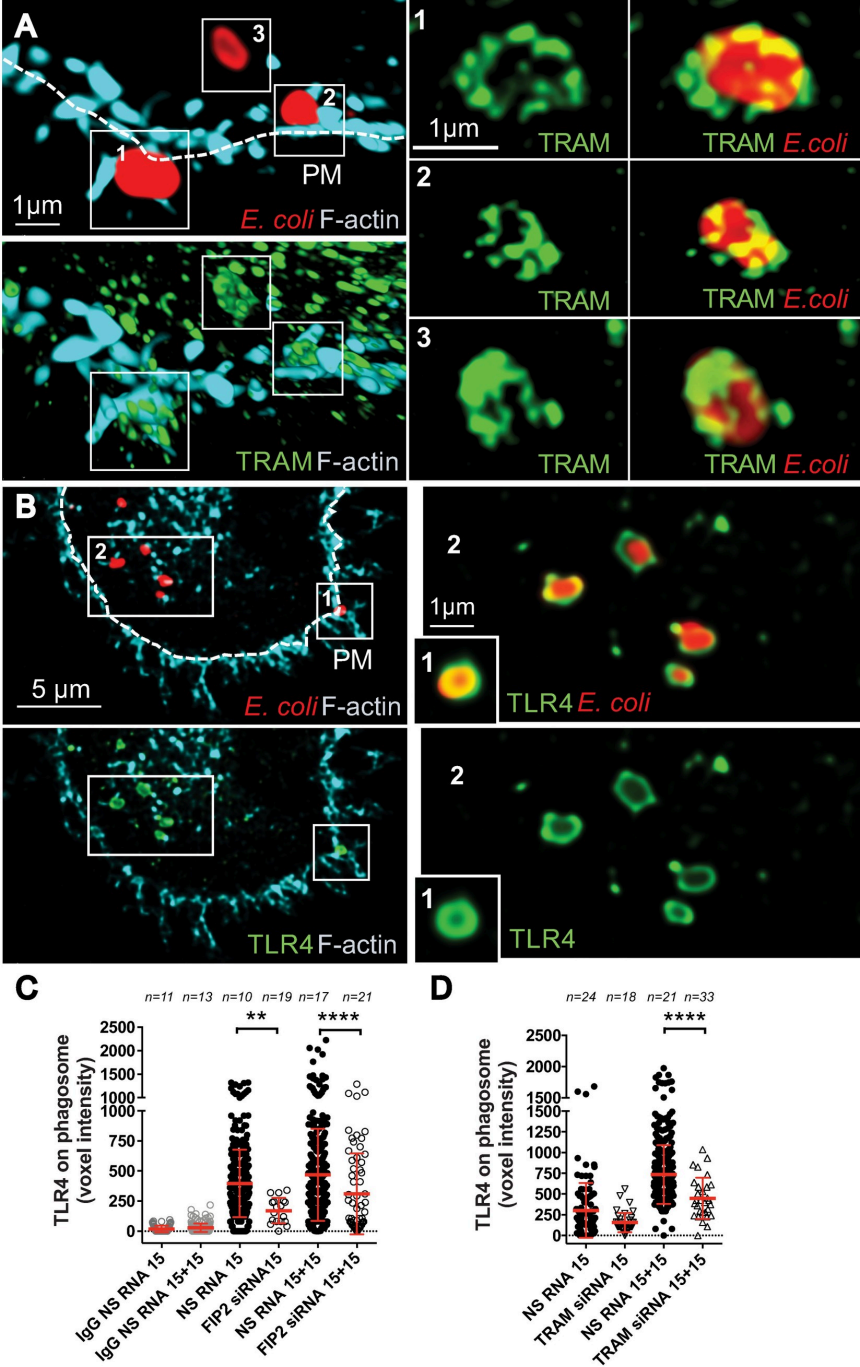
Super resolution microscopy of TRAM and TLR4 at the developing *E*. *coli* phagosome. Primary human macrophages (Mϕ) stimulated with *E*. *coli* bioparticles (red) for 30 min and stained for F-actin (cyan) and TRAM or TLR4 (green) and imaged by 3-D STED. (**A)** Distribution of TRAM before and after internalization of *E*. *coli*. Upper and lower left panels show 3-D rendering of F-actin together with *E*. *coli* or TRAM. Mid and right panels show TRAM alone or TRAM together with *E*. *coli*. (**B)** Distribution of TLR4 before and after internalization of *E*. *coli*. Left panels show F-actin together with *E*. *coli* or TLR4. Right panels show *E*. *coli* together with TLR4 or TLR4 alone. (**C)** TLR4 levels on *E*. *coli* phagosomes in Mϕ treated with NS RNA or FIP2 siRNA and stimulated for 15 and 15+15 min. (**D**) TLR4 levels on *E*. *coli* phagosomes in Mϕ treated with NS RNA or TRAM siRNA and stimulated for 15 min and 15+15 min. n = number of cells monitored. One-way ANOVA Kruskal-Wallis test with adj. p values, **** (p < 0.0001) and ** (p = 0.001). Red bars: mean ± SD from one representative human donor of three. PM = plasma membrane. Scale bars = 1 μm or 5 μm.

To investigate the distribution of TRAM and TLR4 on developing *E*. *coli* phagosomes in detail, 3-D stimulated emission depletion microscopy (3-D STED) was used on primary macrophages stimulated by *E*. *coli* bioparticles for 15+15 min. At 70 nm resolution, TRAM showed a vesicular-tubular pattern towards developing phagosomes (Fig 2A), while TLR4 appeared as an continous envelope (Fig 2B). As *E*. *coli* was internalized, TRAM covered larger parts of the phagosome but still appeared vesicular-tubular, while TLR4 remained mainly as an envelope around the phagosome. As for TRAM, the recruitment of TLR4 to *E*. *coli* phagosomes was significantly reduced in the FIP2 silenced macrophages (Fig 2C). Also, TRAM-silenced macrophages showed a reduction of TLR4 recruitment to *E*. *coli* phagosomes (Fig 2D). Representative images of TLR4 are shown for cells that were treated with NS RNA and FIP2 siRNA or TRAM siRNA and stimulated for 15+15 min with *E*. *coli* (S2A–S2C Fig). The FIP2- and TRAM-silenced macrophages contained significantly less phagosomes and these were frequently located on the plasma membrane. TRAM recruitment to *E*. *coli* during phagocytosis was also verified by live cell imaging of THP-1 cells expressing TRAM-mCherry. Vesicular-tubular TRAM structures were recruited to live *E*. *coli* during internalization, and accumulated as the bacteria entered into the cell (S1 Movie and S2D Fig).

To investigate if FIP2 recruitment to the *E*. *coli* phagosome was a TLR4 dependent process, we included mouse immortalized bone-derived-macrophages (iBMDMs). FIP2 was frequently observed at *E*. *coli* phagosomes after 15 min of stimulation in both wild type and *Tlr4*^*-/-*^ iBMDMs (S3A and S3B Fig). Interestingly, the *TLR4*^*-/-*^ iBMDMs showed significantly reduced FIP2 levels at the *E*. *coli* phagosomes at both 15 and 15+15 min of stimulation (S2C Fig). Together these results demonstrate that TLR4 and TRAM are transported to *E*. *coli* phagosomes by a mechanism involving FIP2.

### In human macrophages TRAM, but not MyD88, is required for *E*. *coli* phagocytosis

**Fig 3:**
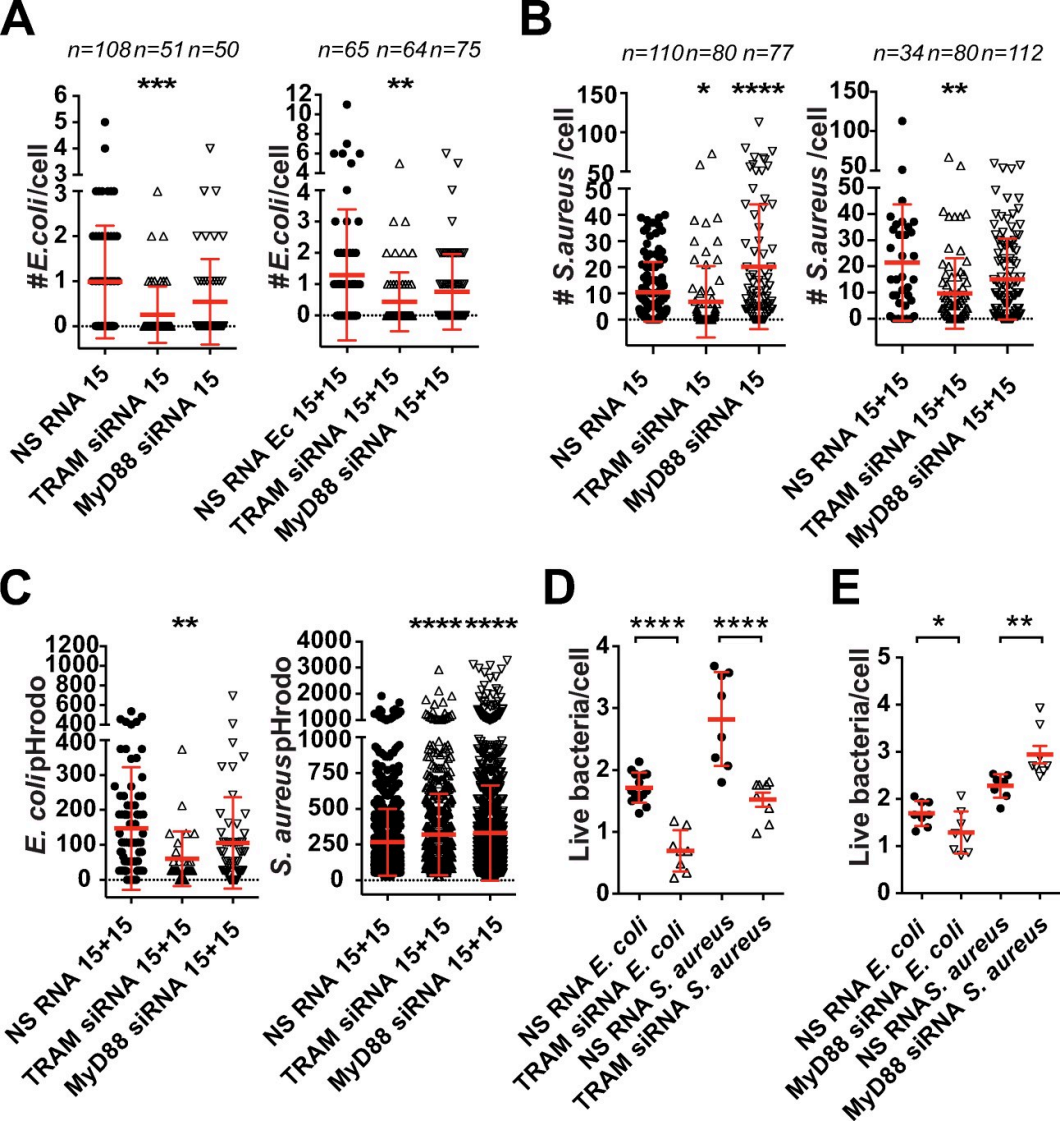
Silencing of TRAM, but not MyD88, inhibits phagocytosis of *E*. *coli* in human macrophages. Human primary macrophages (Mϕ) were treated with NS RNA, TRAM siRNA or MyD88 siRNA and stimulated with *E*. *coli or S*. *aureus* bioparticles for 15 min or 15+15 min. Phagocytosis was monitored by 3-D confocal microscopy and presented as mean bacterial count per cell (**A**) *E*. *coli* phagocytosis in Mϕ stimulated for 15 min or 15+15 min. (**B**) *S*. *aureus* phagocytosis in Mϕ stimulated for 15 min or 15+15 min. (**C**) Phagosome maturation of *E*. *coli*- and *S*. *aureus* phagosomes in the Mϕ stimulated for 15+15 from Fig 3A and 3B. (**D**) Phagocytosis of live *E*. *coli* and *S*. *aureus* in TRAM siRNA treated THP-1 cells. (**E**) Phagocytosis of live *E*. *coli* or *S*. *aureus* in MyD88 siRNA treated THP-1 cells. n = number of cells monitored. One-way ANOVA Kruskal-Wallis test (A-C) or Holm-Sidak´s test (D-E) with adj. P values, ** (p < 0.0027), *** (p = 0.0006), **** (p < 0.0001). Red bars: mean ± SD. Data are representative of three independent experiments.

Because TRAM was found on FIP2 foci containing F-actin on *E*. *coli* phagosomes, we next investigated if TRAM could play a role in phagocytosis. For the study of comparison, we also included *S*. *aureus*. The number of internalized *E*. *coli* and *S*. *aureus* were quantified by 3-D imaging of primary human macrophages silenced for TRAM or MyD88 (Fig 3A and 3B). TRAM silencing reduced the number of phagocytosed *E*. *coli* per macrophage with more than 60% at both investigated time points (Fig 3A). TRAM silencing also affected *S*. *aureus* phagocytosis, particularly after 15+15 min where the reduction was approximately 50% (Fig 3B). MyD88 silencing did not significantly reduce phagocytosis of either *E*. *coli* or *S*. *aureus* (Fig 3A and 3B). Maturation of *E*. *coli* phagosomes was significant impaired by TRAM siRNA, whereas MyD88 silencing had no effect (Fig 3C). Interestingly, both TRAM and MyD88 silenced macrophages showed slight, but significant, increase in *S*. *aureus* phagosome maturation.

We next used THP-1 cells to verify our findings in primary human macrophages, as these cells show more efficient silencing of MyD88 than in primary macrophages (S5A and S5B Fig). Immunoblots of THP-1 cells silenced for MyD88 did not show detectable MyD88 protein (S5C Fig). Moreover, a functional MyD88 control in THP-1 cells confirmed that TNF and IL-6 mRNA expressions were strongly reduced in MyD88 silenced cells stimulated with Pam3CSK4 and LPS (S5D Fig). The effect of TRAM siRNA on the uptake of *E*. *coli* in THP-1 cells was clear and significant and resembled the data obtained with primary macrophages (S5E Fig). Also, flow cytometry analysis showed that TRAM silencing reduced phagocytosis of *E*. *coli* (S5G Fig). In contrast to primary human macrophages, TRAM silencing in THP-1 cells did not significantly reduce the uptake of *S*. *aureus* bioparticles (S5F Fig). Silencing of MyD88 in THP-1 cells did not result in significant reduction in uptake of either *E*. *coli* or *S*. *aureus* (S5E–S5G Fig).

To exclude the possibility that the observed effect of TRAM and MyD88 on phagocytosis, was due to the use of pHrodo-labelled killed bacteria, we also included live bacteria in this study. The phagocytosis of live *E*. *coli* and *S*. *aureus* was measured in a modified phagocytic killing assay [7] and monitored as colony-forming units (cfu) per cell. In line with the phagocytosis data of bioparticles in human macrophages and THP-1 cells, TRAM depleted THP-1 cells showed significant reduction in phagocytosis of live *E*. *coli* (Fig 3D). Of interest, TRAM silencing also reduced uptake of live *S*. *aureus* (Fig 3D). The effect of MyD88 silencing was not so clear with a weak reduction in *E*. *coli* uptake and in fact an increase in phagocytosis of *S*. *aureus* in this assay (Fig 3E).

We next used *Tram*^*-/-*^ and *Myd88*^*-/-*^ iBMDMs and flow cytometry to investigate if mouse macrophages showed a similar phenotype as human macrophages (S5H Fig). While both TRAM and MyD88 deficient mouse macrophages showed impaired phagocytosis of *E*. *coli*, only the MyD88 deficient macrophages reduced *S*. *aureus* phagocytosis. The effect of TRAM- and MyD88 knock out on phagocytosis was also compared with knocking out TLR4. *Tlr4*^-/-^ mouse macrophages showed impaired uptake of *E*. *coli*, however, with less efficiency compared to TRAM- or Myd88-deficient macrophages (S5I Fig). Phagocytosis of *S*. *aureus* was not reduced in the TLR4 deficient macrophages.

Together these results show that TRAM has a strong and consistent phenotype in regulating phagocytosis of *E*. *coli* in human macrophages. TRAM silencing also reduced uptake of *S*. *aureus* bioparticles in primary human macrophages at early timepoints as well as impairing uptake of live bacteria in THP-1 cells. The involvement of MyD88 in phagocytosis of *E*. *coli* and *S*. *aureus* was less clear as differences between the human and mouse macrophages were observed.

### FIP2 and TRAM form a complex that is enhanced by *E*. *coli* stimulation

**Fig 4:**
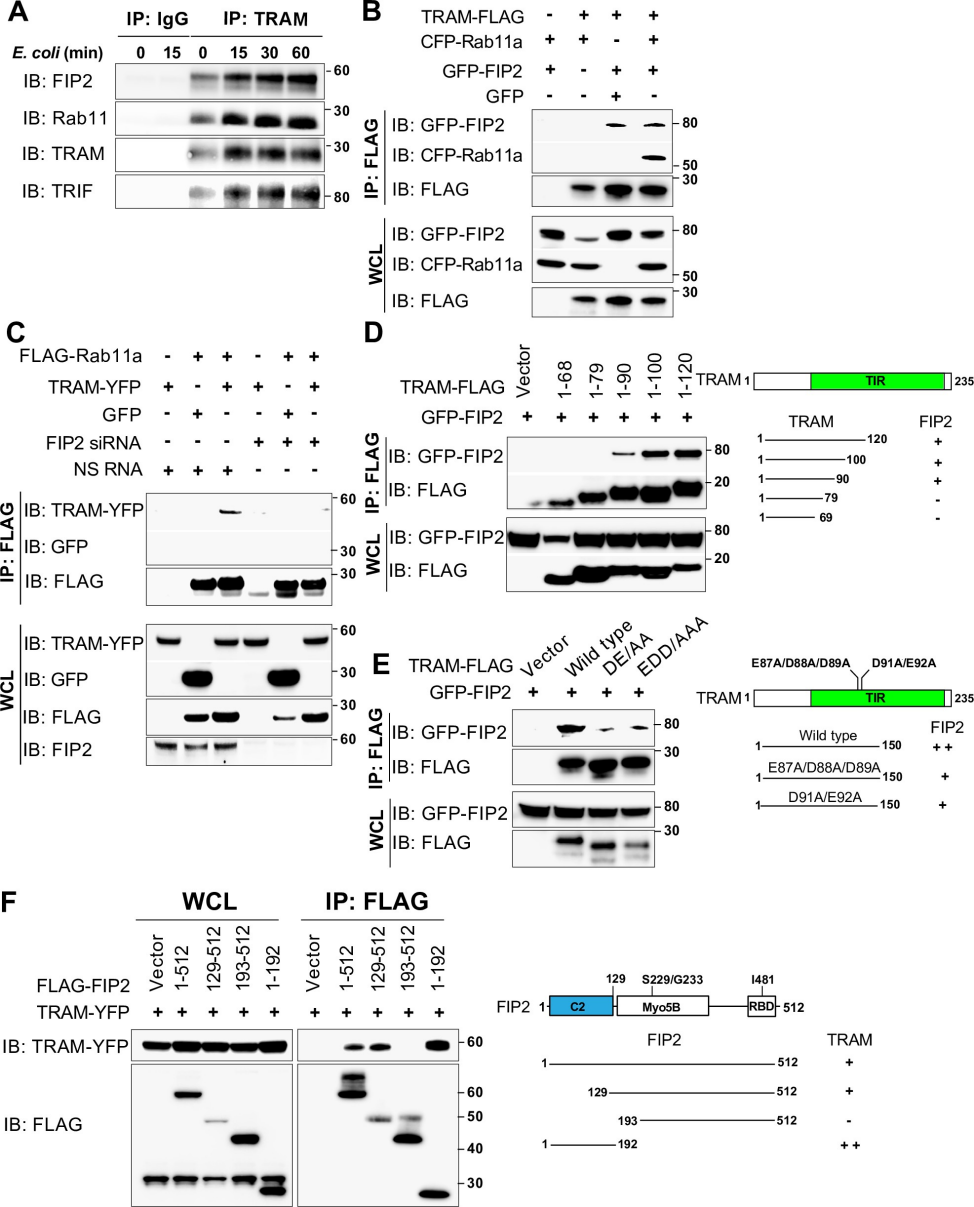
FIP2 forms a complex with TRAM and Rab11a. (**A**) Immunoblot of TRAM immunopreciptitations made from THP-1 cells stimulated with *E*. *coli* bioparticles. TRAM antibody conjugated Dynabeads were used for co-precipitation of FIP2, Rab11 and TRIF from lysates. (**B**) Immunoblot of TRAM-FLAG pulldowns from lysates of HEK293T cells expressing TRAM-FLAG and CFP-Rab11a, GFP-FIP2 or TRAM-FLAG, GFP-FIP2 and GFP. (**C**) Immunoblot of FLAG-Rab11a pulldowns from HEK293T cells treated with NS RNA or FIP2 siRNA expressing FLAG-Rab11a and TRAM-YFP, or GFP. (**D**) Immunoblot of TRAM-FLAG pulldowns from HEK293T cells expressing FLAG-tagged deletion mutants of TRAM (aa 1–68, 1–79, 1–90, 1–100 or 1–120) and EGFP-FIP2. (**E**) Immunoblot of TRAM-FLAG pulldowns from in HEK293T cells expressing FLAG-tagged TRAM (amino acid 1–150), or alanine substitution mutants: TRAM-E87A/D88A/D89A (EDD/AAA) or TRAM-D91A/E92A (DE/AA). (**F**) Immunoblot of FLAG-FIP2 pulldowns from HEK293T cells expressing FLAG-tagged FIP2 wild type or deletion mutants (aa 1–512, 129–512, 193–512 or 1–192). Anti-FLAG M2-agarose was used for pulldown of FLAG-tagged TRAM, FIP2 or Rab11a from lysates as indicated (B-E).

The observation that FIP2 and TRAM co-localized on forming *E*. *coli* phagosomes led us to investigate if TRAM and FIP2 could mutually interact. Indeed, co-immunoprecipitation analyses of THP-1 cells revealed that endogenous TRAM formed a complex with FIP2 (Fig 4A). Interestingly, also Rab11 and TRIF were part of this complex which was markedly increased by *E*. *coli* stimulation. We next co-expressed TRAM and Rab11a in HEK293T cells, with and without FIP2 and found that TRAM and Rab11a formed a complex only when FIP2 was co-expressed (Fig 4B). In line with this result Rab11a and TRAM did not form a complex when endogenous FIP2 was silenced (Fig 4C). Moreover, FIP2 and TRAM could still interact in cells simultaneously silenced for Rab11a and Rab11b (S4A Fig). In support of these results, the FIP2 I481E mutant [21], containing a single amino acid mutation in the Rab11 binding domain of FIP2, could not bind Rab11a, but was found to interact with TRAM (S4B Fig). As expected, FIP2 bound strongly to Rab11a and the constitutive active GTP-bound Rab11aQ70L mutant but did not bind to the inactive GDP-bound Rab11aS25N mutant (S4C Fig). Despite the lack of FIP2 binding to inactive Rab11aS25N, TRAM could still be found in complex with FIP2.

We next used the HEK293 cell model to investigate if FIP2 could be involved in the formation of enlarged LPS endosomes. HEK293 cells expressing human TLR4, CD14, MD2, TRAM and Rab11 form enlarged Rab11 positive endosomes following LPS stimulation [22]. The data demonstrate that FIP2, TRAM and constitutively active Rab11a are present on LPS endosomes (S4D Fig). Cells co-transfected with the inactive form of Rab11a failed to form enlarged LPS endosomes and FIP2 appeared cytosolic (S4E Fig). Taken together, these results suggest that FIP2 controls the localization of TRAM to enlarged LPS endosomes and that active Rab11a is needed for optimal FIP2 binding to TRAM.

To identify the FIP2 binding site in TRAM we analysed a series of TRAM deletion mutants, which contained the N-terminal part of TRAM with 10–20 amino acid residues increments. While TRAM 1–68 and 1–79 did not bind FIP2, a weak interaction was found with TRAM 1–90 that increased markedly with TRAM 1–100, but not further with TRAM 1–120 (Fig 4D). These data show that there is a FIP2 binding site located between the amino acid residues 80–100 in TRAM. This domain contains the acidic amino acid motifs E87/D88/D89 and E91/D92, reported to be required for TLR4-mediated TRAM-TRIF signalling [23, 24]. We next made a TRAM construct with the alanine substitutions E87A/D88A/D89A and D91A/E92A and investigated FIP2 binding. Both E87A/D88A/D89A and D91A/E92A mutants showed noticeably impaired FIP2 binding (Fig 4E).

Next, we made several FLAG-FIP2 variants containing the amino acids residues 1–512 (wild type), and the deletion mutants 129–512, 1–192 and 193–512 to locate the TRAM-binding site in FIP2. While wild type FIP2, FIP2 129–512 and FIP2 1–192 all showed TRAM binding, FIP2 193–512 failed to bind TRAM (Fig 4F). To summarize, we found a sequence of 63 amino acids, located between positions 129–192 of FIP2, to be responsible for TRAM binding. Of interest, FIP2 1–192, which lacks Rab11 and Myosin5B tail binding [25, 26], showed an even stronger binding to TRAM. Taken together these results demonstrate that FIP2 binding to TRAM occurs via FIP2, and not Rab11, but Rab11 positively regulates TRAM-FIP2 complex formation.

### FIP2 controls phagocytosis of both Gram-negative and Gram-positive bacteria

**Fig 5:**
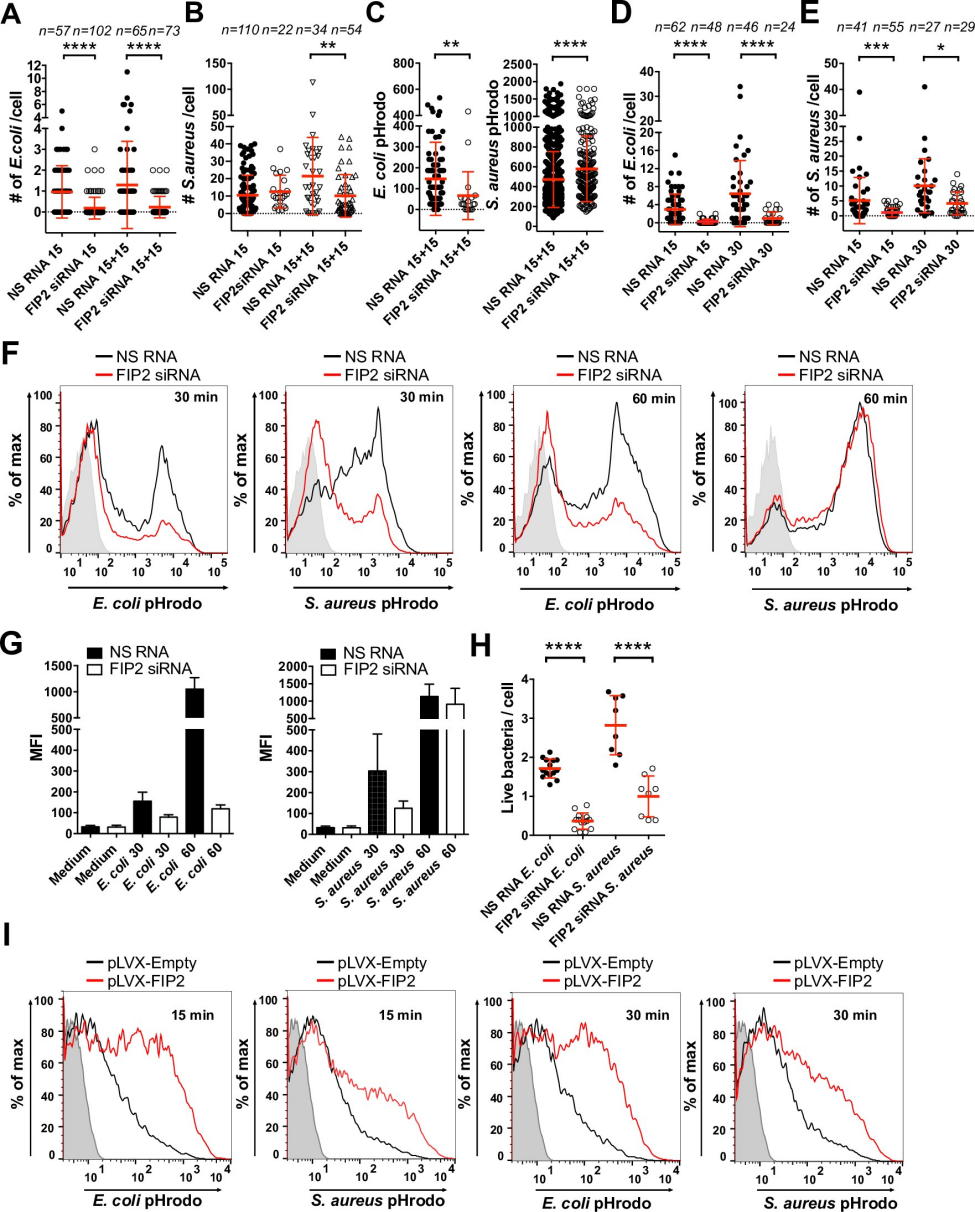
FIP2 is required for phagocytosis. (**A**) *E*. *coli* phagocytosis in FIP2 silenced human primary macrophages. (**B**) *S*. *aureus* phagocytosis in FIP2 silenced human primary macrophages. (**C**) *E*. *coli and S*. *aureus* phagosome maturation in the macrophages from Fig 5A and 5B after 15+15 min of stimulation. (**D**) *E*. *coli* phagocytosis in FIP2 silenced THP-1 cells. (**E**) *S*. *aureus* phagocytosis in FIP2 silenced THP-1 cells. Phagocytosis was monitored by 3-D confocal microscopy and presented as mean bacterial count per cell (A-E). (**F**) *E*. *coli* and *S*. *aureus* phagocytosis in FIP2 silenced THP-1 cells measured by flow cytometry. (**G**) Average mean fluorescence intensity (MFI) from, n = 3, independent experiments with mean ± SEM. (**H**) Phagocytosis of live *E*. *coli* or *S*. *aureus* in FIP2 siRNA treated THP-1 cells. (**I**) *E*. *coli* or *S*. *aureus* phagocytosis in THP-1 cells expressing empty vector (pLVX-Empty) or FIP2 expression vector (pLVX-FIP2) measured by flow cytometry. n = number of cells monitored per condition. One-way ANOVA Kruskal-Wallis test (A, B, D and E) or Holm-Sidak´s test (H) with adj. p values, ** (p< 0.0064), *** (p = 0.0006), **** (p < 0.0001). Red bars: mean ± SD (A-E and H). Data are representative of three or more independent experiments.

**Fig 6:**
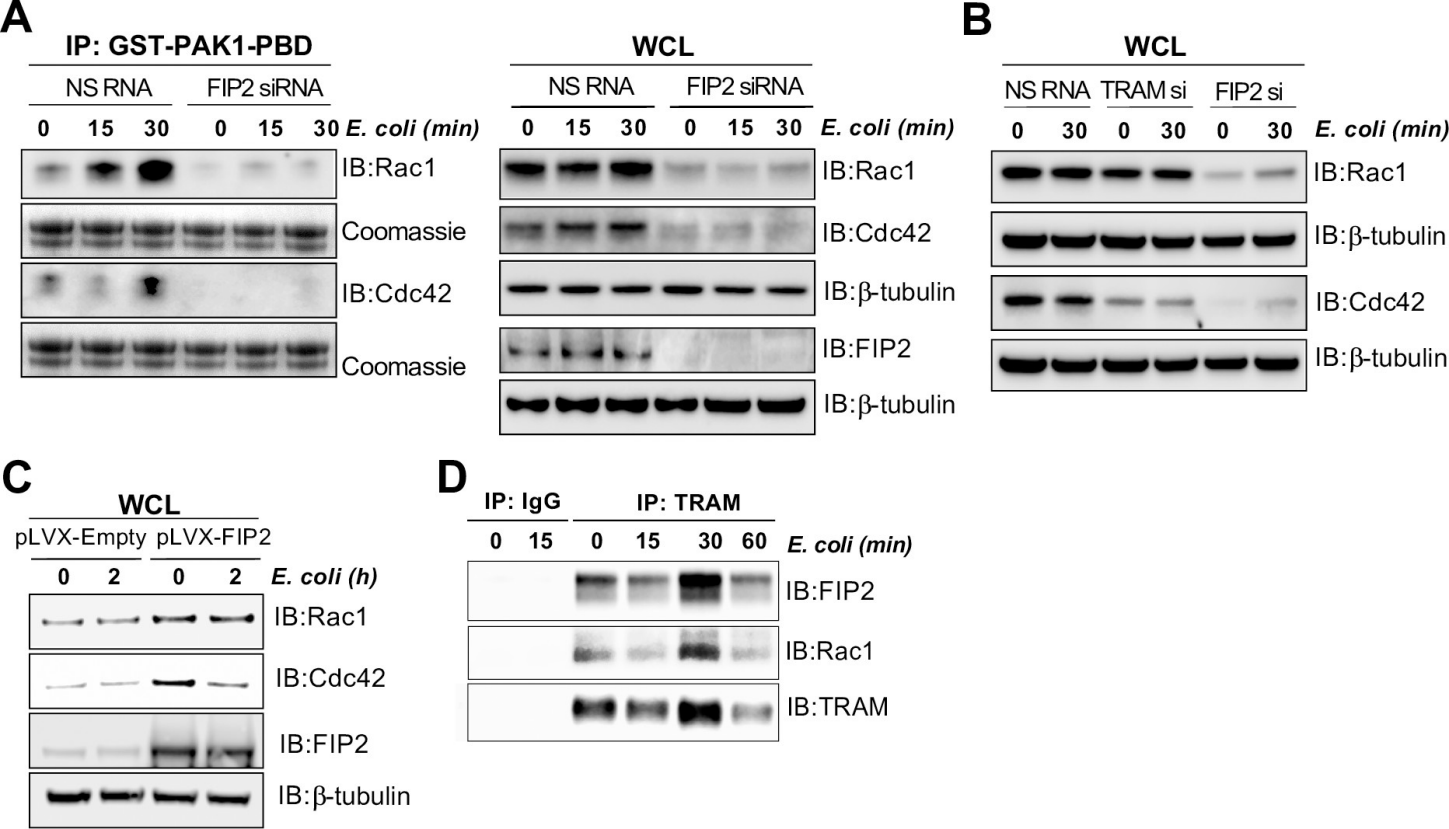
FIP2 controls *E*. *coli* stimulated activation of Rac1 and Cdc42. (**A**) Immunoblot of *E*. *coli* stimulated activation of Cdc42 and Rac1 in THP-1 cells treated with NS RNA and FIP2 siRNA. The activation of Cdc42 and Rac1 was monitored by co-incubating glutathione-agarose beads conjugated with GST-PAK1-PBD with lysates of THP-1 cells stimulated with *E*. *coli* bioparticles as indicated. (**B**) Rac1 and Cdc42 levels in lysates from THP-1 cells treated with NS RNA, TRAM siRNA and FIP2 siRNA. (**C**) Rac1, Cdc42 and FIP2 levels relative to β-tubulin protein levels in wild type THP-1 cells transduced with pLVX-empty- or pLVX-FIP2 vector. (**D**) Immunoblot of primary human macrophages (M) stimulated with *E. coli* bioparticles. TRAM antibody conjugated Dynabeads were used for co-precipitation of FIP2 and Rac1 from lysates.

Since FIP2 interacted with TRAM, and FIP2 silencing specifically decreased *E*. *coli* induced expression of IFN-β (S1A Fig), we next investigated if FIP2 was involved in phagocytosis. Indeed, primary human macrophages silenced for FIP2 showed more than 80% reduction of *E*. *coli* per cell at both 15 and 15+15 min, while *S*. *aureus* phagocytosis was only impaired in cells stimulated for 15+15 min (Fig 5A and 5B). As observed in the TRAM-silenced human primary macrophages, the maturation of *E*. *coli* phagosomes was decreased while the phagosome maturation of *S*. *aureus* phagosomes was increased in FIP2 silenced cells (Figs 3C and 5C). To validate the effect of FIP2 on phagocytosis in primary macrophages, we next used FIP2-silenced THP-1 cells which also showed a marked perturbation of *E*. *coli* and *S*. *aureus* phagocytosis (Fig 5D and 5E). Analysis by flow cytometry showed that FIP2 silencing inhibited phagocytosis of both *E*. *coli* and *S*. *aureus* after 30 min of stimulation, while only *E*. *coli* phagocytosis was reduced after 60 min (Fig 5F and 5G). Also, phagocytosis of live *E*. *coli* and *S*. *aureus* was significantly reduced upon FIP2 silencing (Fig 5H). The effect of FIP2 silencing on the phagocytosis of *E*. *coli* and *S*. *aureus* was comparable to the F-actin inhibitor cytochalasin D (S6C and S6D Fig). In THP-1 cells with lentiviral overexpression of FIP2 a marked increase in both *E*. *coli* and *S*. *aureus* phagocytosis was observed (Fig 5I). As a control, FIP2 overexpression resulted in a strong increase in the amount FIP2 protein (Fig 6C).

Since FIP2 bridges TRAM and Rab11, and complex formation was enhanced by *E*. *coli* stimulation (Fig 4A), we also assessed the role of Rab11 in *E*. *coli* phagocytosis. We have previously shown that recruitment of both TRAM and TLR4 to the *E*. *coli* phagosomes in human macrophages are dependent on Rab11a, however, silencing of Rab11a alone did not affect phagocytosis [10]. To investigate if FIP2 controlled *E*. *coli* phagocytosis via Rab11, we simultaneously silenced the Rab11 isoforms Rab11a and Rab11b in primary human macrophages. Indeed, this resulted in a significant and consistent inhibition of *E*. *coli* phagocytosis similar to the FIP2 and TRAM silenced macrophages (S6E and S6F Fig). These data suggest that redundancy exists between Rab11a and Rab11b and that both isoforms must be targeted in order to affect phagocytosis. Together these results show that FIP2 is an important regulator of phagocytosis of *E*. *coli*. For *S*. *aureus* FIP2 seems to preferentially control phagocytosis at early timepoints, while the effect is lost at 60 min.

### FIP2 controls *E*. *coli* phagocytosis through a mechanism involving TRAM, Rac1 and Cdc42

Rho GTPases, like Rac1 and Cdc42, are instrumental in regulating F-actin dynamics during phagocytosis [3]. Given the strong effect of FIP2 silencing on *E*. *coli* phagocytosis, we analysed Rac1- and Cdc42 activation in these cells. We made a construct encoding the Rac1/Cdc42 (p21) binding domain (PBD) of the human p21 activated kinase 1 protein (PAK) fused to the GST protein. PBD binds specifically to the activated GTP-bound forms of the Rac1 and Cdc42 proteins [27]. We observed that FIP2 silencing had a marked inhibitory effect on *E*. *coli*-induced activation of both Rac1 and Cdc42. Also, we noticed that FIP2 silencing reduced the amounts of Rac1 and Cdc42 proteins, whereas the mRNA levels were unaffected (Fig 6A and S7A Fig). TRAM depletion did not reduce Rac1 protein, but lowered the amount of Cdc42, (Fig 6B), however, both Rac1 and Cdc42 mRNA expressions were significantly reduced by TRAM depletion (S7B Fig). Furthermore, immunoblots of THP-1 cells overexpressing FIP2 showed increased amounts of both Rac1 and Cdc42 suggesting that FIP2 has a stabilising effect on both proteins. Indeed, we found that Rac1 is part of an immune-complex together with TRAM and FIP2 in primary macrophages (Fig 6D). Altogether, these results demonstrate that FIP2 is a central effector molecule of phagocytosis through activation and stabilization of the Rho GTPases Rac1 and Cdc42.

### FIP2 is a regulator of *E*. *coli* induced TLR4-TRAM-TRIF signalling

**Fig 7:**
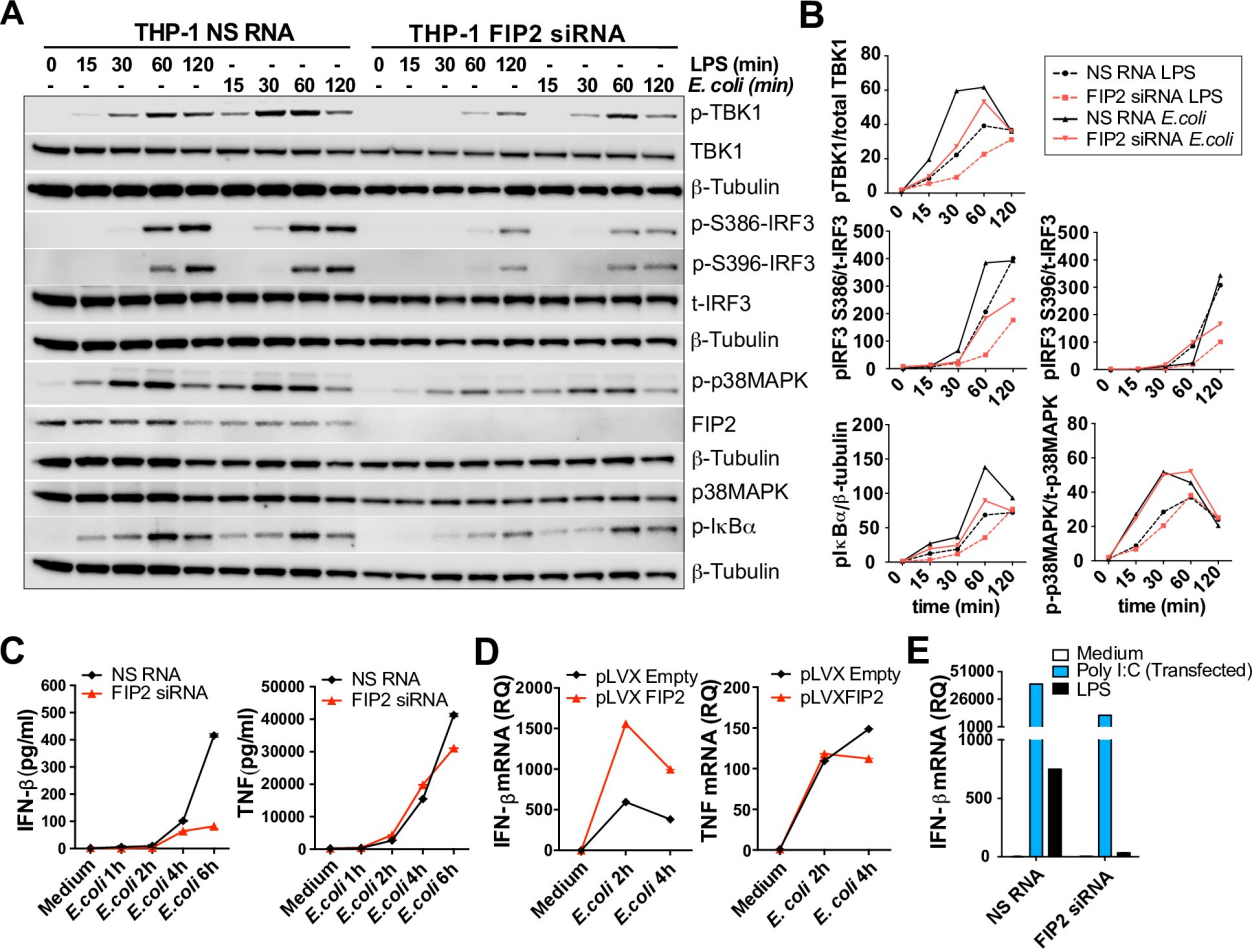
FIP2 controls *E*. *coli* induced IFN-β mRNA induction and secretion. (**A**) Immunoblots showing the phosphorylation patterns of TBK1, IRF3, IκBα and p38MAPK in FIP2 silenced THP-1 cells stimulated with *E*. *coli* bioparticles or LPS (100 ng/ml). Data are representative of three independent experiments. (**B**) Quantification of phosphorylation patterns of the proteins shown in the immunoblots presented in (A). (**C**) ELISA quantification IFN-β and TNF secretion in THP-1 cells treated with NS RNA or FIP2 siRNA and stimulated as indicated. (**D**) Quantification of *E*. *coli*-stimulated IFN-β and TNF mRNAs in THP-1 cells with lentiviral overexpression of FIP2. (**E**) Quantification of Poly I:C and LPS stimulated IFN-β mRNA induction in cells treated with NS RNA or FIP2 siRNA after 4 hours of stimulation. Poly I:C (5 μg/ml) was transfected using Lipofectamine® 2000. Data are representative of three independent experiments.

As FIP2 was found to be a key regulator of *E*. *coli* phagocytosis, we would expect a decreased TRAM-TRIF signalling upon FIP2 depletion with siRNA. Thus, we examined how LPS- and *E*. *coli*-stimulated signalling was affected in FIP2 silenced THP-1 cells (Fig 7A). Following stimulation, phosphorylation of the TANK-binding kinase-1 (TBK-1), IRF3 and IκBα were quantified and found to be markedly impaired in the FIP2 silenced cells, while the phosphorylation of p38 mitogen-activated protein kinase (p38 MAPK) was not markedly impaired (Fig 7A and 7B, S8A Fig). Similar results were obtained using LPS for stimulation. Phosphorylation of TBK-1 at Ser172, IRF3 at Ser386 and Ser396, are all critical for IRF3 activation and induction of IFN-β [28, 29]. In line with the phosphorylation patterns observed by Western blotting, the FIP2 silenced cells showed a markedly impaired induction of IFN-β with little effect on TNF (Fig 7C). THP-1 cells with lentiviral-induced overexpression of FIP2 showed a 3-fold higher *E*. *coli*-stimulated IFN-β mRNA expression (Fig 7D). In contrast, *E*. *coli-*stimulated TNF expression was relatively unchanged by FIP2 overexpression (Fig 7D).

Next, we investigated how the FIP2 silenced cells responded upon MDA5/RIG-I activation that also uses IRF3 to induce IFN-β mRNA expression. The cells were stimulated by poly I:C using lipofectamine transfection and LPS was included for comparison. When transfected, poly I:C triggers a TLR3-independent IRF3-mediated induction of IFN-β via cytosolic receptors MDA5 and RIG-I. MDA5/RIG-I stimulated IFN-β mRNA expression was reduced by only 3.5-fold, while TLR4 stimulated IFN-β mRNAs by LPS was reduced 22-fold after 4 h of stimulation (Fig 7E). Taken together, these data demonstrate that FIP2 is a master regulator of LPS*-* and *E*. *coli-* mediated TLR4-TRAM-TRIF signalling, in addition to being a critical regulator of phagocytosis.

### TBK1 activation is required for phagocytosis of *E*. *coli*

Next, we addressed if inhibition of TLR4-mediated TRAM-TRIF signalling could alter macrophage *E*. *coli* phagocytosis. The TBK1 kinase operates downstream of TRIF and its activity is required for LPS-stimulated phosphorylation of IRF3 and production of IFN-β [28, 29]. First, Western blot analysis was performed in THP-1 cells in order to compare the effect of two TBK1 inhibitors BX-795 and MRT67307 on *E*. *coli*-stimulated IRF3- and p38 MAPK-activation. Both inhibitors impaired IRF3 phosphorylation at Ser386 by more that 65% after 30 min of stimulation (S8B and S8C Fig). In contrast, phosphorylation of p38MAPK was largely unchanged. Next, THP-1 cells and human primary macrophages were treated with the TBK1 inhibitors prior to addition of *E*. *coli* bioparticles. In cells with inhibited TBK1 activity, a marked reduction of phagocytosis of *E*. *coli* was observed after 15 min of stimulation (S8D Fig). When comparing *E*. *coli* and *S*. *aureus* phagocytosis in MRT67307 treated THP-1 cells we found only *E*. *coli* phagocytosis to be significantly decreased (S8E Fig). Also, when TBK1 was inhibited in primary human macrophages a significant and marked reduction of *E*. *coli* phagocytosis was observed (S8F Fig). These results demonstrate that the early phagocytosis of *E*. *coli*, but not *S*. *aureus*, can be targeted by TBK1 kinase inhibition without affecting p38 MAPK activation.

### FIP2 is instrumental for IRF3 target genes induced by *E*. *coli*

**Fig 8:**
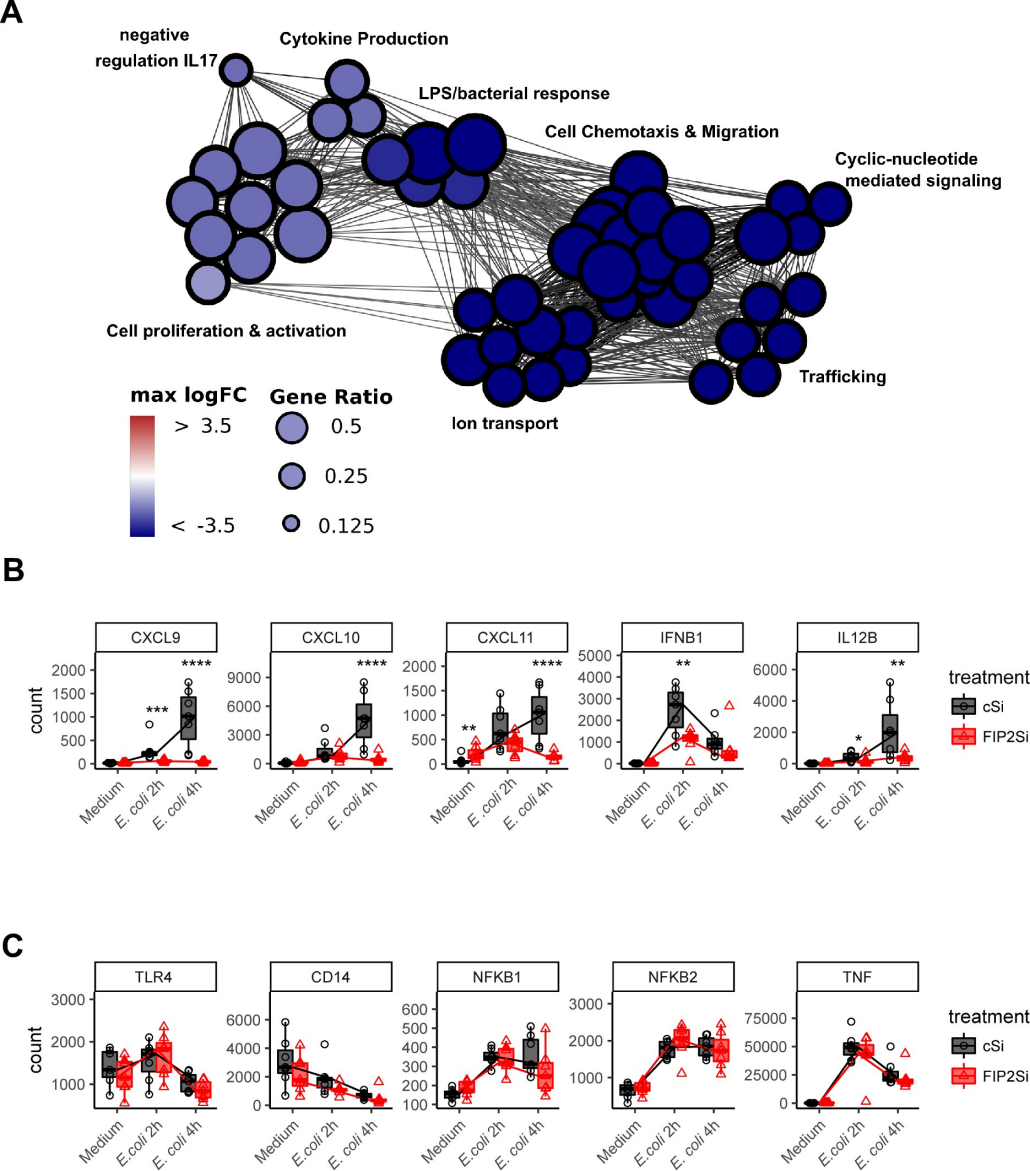
The involvement of FIP2 in human macrophage biology. (**A**) Gene Ontology mapping showing the effect of FIP2 silencing on biological processes in primary human macrophages from 7 donors. (**B**) Effect of FIP2 silencing on *E*. *coli*-stimulated induction of IRF3-target genes. (**C**) Effect of FIP2 silencing on the *E*. *coli*-stimulated induction of a selection of pro-inflammatory genes. Data are median counts with boxed 1.5 IQR 95%. **** (FDR< 0.0001), *** (FDR<0.001), ** (FDR<0.01), * (FDR<0.05). FDR = False Discovery Rate, IQR = Inter Quantile Range.

In order to examine the importance of FIP2 on bacterially induced gene regulation, we performed a targeted transcriptome profiling for immunologically relevant genes on RNA samples isolated from *E*. *coli* stimulated human macrophages from 7 donors. Hyper geometric Gene Ontology enrichment for biological processes was performed on genes differentially expressed during *E*. *coli* stimulation in FIP2 silenced cells. When compared to non-silenced macrophages, we found significant hits on downregulated genes involved in several cellular processes essential for innate immunity (Fig 8A). FIP2 depletion had a modest effect on cytokine production, proliferation and activation of macrophages, and most prominent effects on genes regulating the LPS/bacterial stimulated responses, cell chemotaxis & migration, cyclic nucleotide mediated signalling, ion transport and intracellular trafficking (Fig 8A). After 4 h of *E*. *coli* stimulation, the chemokines CXCL9, CXCL10 and CXCL11, together with IL12B (IL12p40) were among the most downregulated genes in the FIP2 silenced macrophages (Fig 8B and S3 Table). *E*. *coli*-stimulated IFN-β mRNA expression was at its highest after 2 h of stimulation and was among the 7 most downregulated genes after FIP2 silencing at this time point (Fig 8B and S2 Table). In contrast, FIP2 silencing did not significantly alter *E*. *coli*-stimulated mRNA expression of TLR4, CD14, NF-κB1, NF-κB2 and TNF (Fig 8C and S1–S3 Tables). The results from the Nanostring experiment were verified by qPCR of selected cytokines. These data confirmed that IFN-β, CXCL9, CXCL10, CXCL11 and IL12B were significantly reduced in FIP2 silenced macrophages, while TNF, TLR4 and CD14 were not changed (S9A and S9B Fig). The result from these experiments demonstrate that FIP2 silencing has a preference for reducing TLR4-stimulated induction of IRF3 target genes, which is likely to be a consequence of the impaired phagocytosis of *E*. *coli*.

## Discussion

In the present study, we show that TLR4 mediates phagocytosis of *E*. *coli* in macrophages via its adaptor TRAM. TRAM performs this function by interacting with FIP2 which subsequently activates the Rac1 and Cdc42 Rho GTPases for controlling actin-dynamics. A consequence of this is that FIP2 strongly regulates phagosomal signalling that involves IRF3 activation. Receptor recognition during phagocytosis launches signalling pathways that induce remodelling of the actin cytoskeleton and extension of membrane protrusions that surround the particle to form a phagocytic cup [30]. In early phases of *E*. *coli* phagosome formation, TLR4 is recruited to the phagocytic cup to provide a platform for subsequent TRAM-TRIF signalling [10]. Our findings demonstrate that TRAM recruitment to this platform requires FIP2 and that TLR4-TRAM-TRIF signalling is needed for phagocytosis.

MyD88 is a universal signalling adaptor for TLRs, except TLR3, and activates NF-kB, c-Jun kinase, and p38 MAPK [31]. In mouse macrophages, Blander and co- workers found that MyD88-mediated signalling is required for phagocytosis of *E*. *coli* and *S*. *aureus* and for phagosomal maturation [7]. In contrast to these findings, Yates and Russel showed that the phagosome maturation of beads coated with the TLR4 ligand LPS or the TLR2 ligand Pam3Cys occurs independently of MyD88-mediated signalling [32]. The controversy on the involvement of MyD88 in phagosomal maturation in murine macrophages may be due to variations in experimental models used. In previous studies on murine macrophages, the involvement of TRAM-TRIF signalling in phagocytosis and phagosomal maturation has not addressed. Our data suggest that in human macrophages TRAM, but not MyD88, is involved in both uptake of *E*. *coli* as well as in phagosomal maturation. We found that murine macrophages deficient for TRAM or MyD88 showed a markedly reduced uptake of *E*. *coli* whereas only MyD88 affected phagocytosis of *S*. *aureus*. Apparently, mouse macrophages use both MyD88-dependent and MyD88-independent signalling for controlling phagocytosis of *E*. *coli*, whereas only MyD88 played a role for the uptake of *S*. *aureus*. The role of MyD88 in phagocytosis agrees with the data from murine macrophages published by Blander and co-workers [7]. The mechanism behind the species differences between human and mouse macrophages regarding the role of MyD88 in phagocytosis of Gram-positive bacteria are not clear. It has been shown that phagosomes in murine M1 macrophages become more acidic in mice compared to M1 macrophages in humans [33–35]. Thus, murine and human macrophages may behave differently in phagocytic processes.

We observed that silencing of TRAM in fact reduced the uptake of both heat-killed *S*. *aureus* and *E*. *coli* in primary human macrophages, and both heat-killed and live bacteria in THP-1 cells. Several explanations may account for this effect of TRAM on phagocytosis. It is known that lipoproteins and lipoteichoic acids present in Gram-positive bacteria interact with TLR2 [36]. TLR2 can mediate signal transduction through TRAM-TRIF and IRF3, in addition to MyD88 and IRF1 [37]. Also, TRAM has been reported to act as a bridging adapter with MyD88 to control TLR2-mediated induction of IFN-β via IRF7 [38]. These previous reported TLR2-dependent TRAM responses were observed after prolonged stimulation, whereas heat killed *E*. *coli*-stimulated IRF3 activation occurred rapidly within 30 min. The role of TRAM in phagocytosis of *S*. *aureus* may be uncoupled to TLR2 signalling. This is supported by our TBK-1 inhibitor data showing that phagocytosis of heat killed *S*. *aureus* is not reduced, whereas heat killed *E*. *coli* uptake was significantly inhibited. This may implicate that TRAM-TRIF signalling is linked to *E*. *coli*, but not *S*. *aureus*, phagocytosis. Moreover, TRAM was strongly recruited to heat killed *E*. *coli*, but not *S*. *aureus*, phagosomes. Furthermore, silencing of TRAM reduced *E*. *coli* phagosome maturation, but had no inhibitory effect on *S*. *aureus* phagosomes. Of interest, we found that TRAM silencing reduced mRNA expression of both Rac1 and Cdc42 as well as reducing the amount of Cdc42 protein. Given the fact that RhoGTPases are so instrumental in actin dynamics we suggest that TRAM may regulate phagocytosis of both *E*. *coli* and *S*. *aureus* by controlling the levels of Rac1 and Cdc42 in macrophages.

FIP2 was found to be a master regulator of *E*. *coli* uptake. Phagocytosis of *S*. *aureus* was also reduced by FIP2 silencing, however, the effect seemed weaker and appeared to be lost after 60 min. Overexpression of FIP2 increased markedly the internalization of both *E*. *coli* and *S*. *aureus*. Silencing of FIP2 lead to decreased amounts of activated Rac1 and Cdc42 as well as reduced amounts of the proteins, without affecting their mRNA expression levels. Conversely, overexpression of FIP2 in THP-1 cells resulted in an increase in Rac1 and Cdc42 proteins. We suggest that FIP2 controls the RhoGTPases through ubiquitination and proteasomal degradation. Several studies have shown that Rho GTPases are regulated by post-translational modifications such as ubiquitination [39–41]. Our data also suggest that Rac1 may be stabilized through its interaction with FIP2 and TRAM. The profound effect of FIP2 on Rac1 and Cdc42 stability will have important functional consequences on actin dynamics and phagocytosis. This statement is supported by our data demonstrating that FIP2 silencing markedly reduces F-actin and TRAM on *E*. *coli* phagosomes. Moreover, data from Dong et al [42] have shown that FIP2 affects actin cytoskeleton dynamics in cancer cells, however, the mechanisms behind this effect was not addressed in their study. Since FIP2 is regulating both activation and stability of Rac1 and Cdc42 it is conceivable that it controls phagocytosis of heat-killed and live *E*. *coli* and *S*. *aureus* bacteria.

In our experiments we have used siRNA technology to deplete TRAM and FIP2. We made several THP-1 knock out cell lines using CRISP/Cas9 technology targeting TRAM and FIP2. However, we experienced problems with these cell lines related to stability and compensatory mechanisms. Thus, we found it more reliable to reduce gene expression by siRNA, instead of using the CRISPR/Cas9 technology, which allowed comparison of the THP-1 cell system with primary human macrophages.

TRAM can interact with proteins that do not contain a TIR domain [43]. In a recent study we reported that SLAMF1 binds to TRAM and regulates its transport to *E*. *coli* phagosomes and IFN-β release but does not affect phagocytosis [44]. In the current paper we show that TRAM also interacted with FIP2. The binding of FIP2 to TRAM was not dependent on Rab11, however, Rab11 was found to be a part of the FIP2-TRAM complex. Moreover, TLR4 activation increased the amount of endogenous FIP2 and TRAM complexes suggesting that TLR4 may augment FIP2-TRAM interaction by activation of Rab11.

The domain in TRAM involved in interaction with FIP2 was mapped to the amino acid residues 80–100. Structural analysis shows that human TRAM and TRIF form a BB-loop–mediated homodimer at amino acid residues P116 and C117, critical for TRAM and TRIF dimerization and subsequent signalling [23, 45]. Moreover, Funami and co-workers reported that the E87/D88/D89 motif in TRAM is indispensable for TRAM-TRIF dimerization while the D91/E92 motif is not [24]. Our data demonstrate that both the E87/D88/D89 and D91/E92 motifs are critical for FIP2 interaction. The fact that the complex formation between TRAM, FIP2, Rab11 and TRIF was increased by TLR4 stimulation suggest that FIP2 does not interfere with binding of TRIF to TRAM. Furthermore, we located the TRAM binding domain in FIP2, between the amino acid residues 129–192, that does not contain the motifs required for Myosin5B or Rab11 binding [25, 26]. Our data suggest that the interaction between TRAM and FIP2 is required both for uptake of *E*. *coli* and for phagosomal maturation in primary human macrophages. In these cells Rac1 was found to be in complex with FIP2 and TRAM which may explain the close relationship with this complex and phagocytosis. In summary, we describe a novel function of TRAM in the regulation of phagocytosis of Gram-negative bacteria. Our model suggests that FIP2 exists in a preformed complex with TRAM-TRIF that is rapidly recruited to the *E*. *coli* binding site and enhanced by TLR4 activation. This allows FIP2 to activate Rac1 and Cdc42 resulting in F-actin formation at the phagocytic cup which together with TLR4-mediated TRAM-TRIF signalling is required for uptake of the bacteria.

## Materials and methods

### Reagents and bacteria

The following ligands, bacteria and inhibitors were used: pHrodo Red *E*. *coli* K12 BioParticles (P35361), pHrodo Red *S*. *aureus* BioParticles (A10010) from Invitrogen, *E*. *coli* K12 (DH5α) and *S*. *aureus* (ATCC® 10832D-5™). Ultrapure LPS from *E*. *coli* K12 (tlrl-eklps) and Poly I:C HMW (tlrl-pic) from InvivoGen. Live DH5α *E*. *coli* expressing pZE27GFP was a gift from James Collins (Addgene plasmid 75452). TBK1 inhibitors MRT67307 and Bx-795 from Sigma-Aldrich.

### Cells and cell lines

THP-1 cells (monocytic cell line derived from acute monocytic leukemia ATCC® TIB-202™) was maintained in RPMI-1640 (ATCC® 30–2001™) complemented with 2-mercaptoethanol to 0.05 mM and 10% fetal calf serum (FCS) (10270106 GIBCO) at 37 ^o^C and 5% CO_2_. THP-1 cells were differentiated in growth medium supplemented with 40–60 ng/mL phorbol 12-myristate 13-acetate (P8139 Sigma-Aldrich). Human monocytes were isolated from buffy coats (Department of Immunology and Transfusion Medicine, St Olavs Hospital) and differentiated into macrophages in RPMI1640 supplemented with 50 ng/mL recombinant human M-CSF (216-MC-025 R&D systems), 10% pooled human A+ serum (Department of Immunology and Transfusion Medicine, St Olavs Hospital), 700 μM L-glutamine (Sigma-Aldrich) and 20 μg/mL Gensumycin (Sanofi-Aventis) at 37 ^o^C and 5% CO_2_. Medium was changed on day 3 and 5 after seeding. HEK293T cells (Human epithelial cells ATCCCRL-11268) and HEK293-TLR4^mCherry^ cells (were made by us as described in [10]) and maintained at 37 ^o^C and 8% CO_2_ in DMEM supplemented with 10% FCS, 1 μg/mL of Ciprofloxacin Hydrochloride (CellGro®). 0.5 mg/mL G418 (Geneticin, Life Technologies) were used for TLR4^mCherry^ selection. Transfection of plasmids was performed using GeneJuice transfection reagent (Novagen). The iBMDMs (Immortalized bone-derived-macrophages) from wild type, *Tlr4*^*-*^*/*^*-*^, *Tram*^-/-^ and *Myd88*^-/-^ C57BL/6 mice were made in the lab of Dr. Douglas T. Golenbock [46] and maintained as the HEK293T cells above.

### Stimulation of cells

pHrodo-conjugated *E*. *coli* or *S*. *aureus* heat killed bacterial bioparticles were given to the cells in doses ranging from 7.5 to 65 particles per cell dependent on the cellular assay. Prior stimulation both LPS and the bacterial particles were sonicated and opsonized in medium containing 10% human A^+^ serum for 5 min at 37 ^o^C. The LPS dose was 100 ng/mL. 5 ug/ mL Poly I:C was transfected with Lipofectamine®RNAiMAX. Live *E*. *coli* and *S*. *aureus* were grown to a density of OD_600_ = 0.35, washed with PBS and given at a dose of 10–50 bacteria per cell.

### siRNA treatment

THP-1 or HEK293T cells were seeded 24 h before siRNA transfection at a density of 400 000–500 000 cells /well in 6-well plates (NUNC) in their respective growth medium containing no antibiotics. siRNA was transfected at a concentration of 16 nM or 8 nM using Lipofectamine®RNAiMAX transfection reagent (Invitrogen) for 48–72 h. PBMC derived macrophages were transfected with 32 nM siRNA on day 6 and 8 after seeding using Lipofectamine®3000 Transfection Reagent (Invitrogen). Medium was changed to fresh antibiotic-free medium 2 h before the second siRNA transfection and the cells stimulated on day 10. The AllStars Negative Control siRNA (SI03650318 QIAGEN) was used as a non-silencing control and termed NS RNA. Hs_RAB11A_5, Hs_RAB11B_6, Hs_RAB11FIP1_12, Hs_RAB11FIP2_5, Hs_Rab11FIP3_9, Hs_RAB11FIP4_5, Hs_RAB11FIP5_5, Hs_TICAM2_2 and Hs_MyD88_5 validated siRNA, all from QIAGEN, were used to target Rab11a, Rab11b, Rab11FIP1, Rab11FIP2, Rab11FIP3, Rab11FIP4, Rab11FIP5, TRAM and MyD88 mRNA, respectively.

### Generation of a stable THP-1 cell line overexpressing FIP2 by lentiviral transduction

THP-1 expressing lentiviral encoding FIP2 was made by cloning FIP2 into the bicistronic lentiviral expression vector pLVX-EF1α-IRES-ZsGreen1 (Clontech) and co-transfect with packaging plasmids psPAX2 and pMD2.G, kindly provided by the TronoLab (Addgene plasmids 12260 and 12259, to produce pseudoviral particles in HEK293T cells. Supernatants were collected at 48 h and 72 h, combined and concentrated using Lenti-X™ Concentrator (Clontech). Subsequently, the viral particles were used for transduction of THP-1 wild type cells along with virus particles without FIP2 coding sequence and ZsGreen positive cells selected by fluorescence-activated cell sorting (FACS) and tested for FIP2 protein expression by Western blotting.

### Generation of a stable THP-1 cell line expressing TRAM^mCherry^

A THP-1 cell line expressing TRAM^mCherry^ were generated using lentiviral transduction. TRAM^mCherry^ was first subcloned into a Gateway ENTRY vector, before recombination into pCDH-EF1a-GW-IRES-Puro [47] from this vector the constructs was packaged into lentivirus particles using third-generation packaging system, a gift from the TronoLab (Addgene plasmids 12251, 12253 and 12259) and according to [48]. Transduced cells were selected using puromycin (1 μg/mL).

### Gene expression analysis

Total RNA was isolated from THP-1 cells or PBMC derived macrophages using QIAzol (Qiagen) or Isol (5 prime) and chloroform extraction followed by purification on RNeasy Mini columns, including DNAse digestion (Qiagen). cDNA was made from total RNA with Maxima First Strand cDNA Synthesis Kit for RT-qPCR (Thermo Scientific). Quantitative real-time PCR (q-PCR) was performed with the PerfeCTa qPCR FastMix (Quanta Biosciences) in 20 μL reaction volume in duplicate wells and cycled in a StepOnePlus™ Real-Time PCR cycler (Applied Biosystems). The following TaqMan Gene Expression Assays (Applied Biosystems) were used: IFN-β (Hs01077958_s1), TNF (Hs00174128_m1), Rab11a (Hs00900539_m1), Rab11b (Hs00188448_m1), Rab11FIP1 (Hs00951195_m1), Rab11FIP2 (Hs00208593_m1), Rab11FIP3 (Hs006085_m1), Rab11FIP4 (Hs00400200_m1), Rab11FIP5 (Hs00392033_m1), TBP (Hs00427620_m1), Rac1 (Hs00251654_m1), Cdc42 (Hs00741586_mH), CXCL9 (Hs00171065_m1), CXCL10 (Hs01124251_g1), CXCL11 (Hs04187682_g1), IL12B (Hs01011518_m1), TLR4 (Hs00152939_m1), CD14 (Hs02621496_s1), IL6 (Hs00985639_m1) and GAPDH (Hs99999905_m1). The level of TBP or GAPDH mRNA was used for normalization and results presented as relative expression compared to the control-treated sample. Relative expression was calculated using the Pfaffl's mathematical model [49].

### ELISA

TNF in supernatants from THP-1 cells was detected using human TNF-alpha DuoSet ELISA (DY210-05 R&D Systems), IFN-β by VeriKine-HSTM Human Interferon-Beta Serum ELISA Kit (41410 PBL Assay Science).

### Cloning of expression constructs

Phusion High-Fidelity DNA Polymerase (Thermo Fisher Scientific) was used for amplification of desired gene sequences. PCR products, or restricted vectors, were purified by QIAquick PCR purification and gel extraction kits (QIAGEN). Endofree plasmid Maxi kit (QIAGEN) was used for endotoxin-free plasmids preparations. Sequencing of plasmids was done at Eurofins Genomics. Primers used for cloning are listed in Table 1. pEGFP-FIP2 (KIAA0941 sequence in pEGFP-C1) and pEGFP-FIP2ΔC2 [50], FIP2 I481E [21] were used as templates. FIP2 and deletion mutants were subcloned into pCMV-(DYKDDDDK)-N vector (Clontech). pLVX-EF1α-IRES-ZsGreen-FIP2 was made by restriction digest of the vector with EcoRI and ligation with EcoRI fragment from pEGFP-FIP2. pcDNA3-hTRAM-YFP was a gift from K. Fitzgerald (University of Massachusetts Medical School, Worcester, MA, USA), used for transfections or as template for subcloning of TRAM and TRAM deletion mutants into pCMV-(DYKDDDDK)-C Vector (Clontech).

**Table 1:** Primers, templates and vectors used for cloning of FIP2 and TRAM mutants.

Construct	Template	Primer sequence 5’-3’		Restriction site	Vector
FIP2 constructs (hRab11FIP2 KIAA0941)					
FIP2	pEGFP-FIP2	Fwd	GCCCGAATTCGGCTGTCCGAGCAAGCCCAAAAG	EcoRI	N-terminal DYKDDDDK (Flag)
		Rev	ATAGCGGCCGCTCATTAACTGTTAGAGAATTTGCCAGC	NotI	
FIP2 1–192	pEGFP-FIP2	Fwd	GCCCGAATTCGGCTGTCCGAGCAAGCCCAAAAG	EcoRI	
		Rev	ATAGCGGCCGCTTAGTGAGTACTTGGAATGATTGC	NotI	
FIP2 129–512	pEGFP-FIP2	Fwd	GCCCGAATTCGGCGAATCAAAAACAGGGGTGAG	EcoRI	
		Rev	ATAGCGGCCGCTCATTAACTGTTAGAGAATTTGCCAGC	NotI	
FIP2 193–512	pEGFP-FIP2	Fwd	GCCCGAATTCGGATGCCCGATGCCAATAGTGAA	EcoRI	
		Rev	ATAGCGGCCGCTCATTAACTGTTAGAGAATTTGCCAGC	NotI	
FIP2 I481E	pEGFP-FIP2 I481E	Fwd	GCCCGAATTCGGCTGTCCGAGCAAGCCCAAAAG	EcoRI	
		Rev	ATAGCGGCCGCTCATTAACTGTTAGAGAATTTGCCAGC	NotI	
TRAM constructs (hTICAM-2 NM_021649.7)					
TRAM	TRAM-YFP	Fwd	CATGAATTCATGGGTATCGGGAAGTCTAAA	EcoRI	C-terminal DYKDDDDK (Flag)
		Rev	TTAACTCGAGCGGCAATAAATTGTCTTTGTACC	XhoI	
TRAM 1–68	TRAM-YFP	Fwd	CATGAATTCATGGGTATCGGGAAGTCTAAA	EcoRI	
		Rev	TTAACTCGAGCCATCTCTTCCACGCTCTGAGC	XhoI	
TRAM 1–79	TRAM-YFP	Fwd	CATGAATTCATGGGTATCGGGAAGTCTAAA	EcoRI	
		Rev	TTACCTCGAGAGAGGAACACCTCTTCTTCAGC	XhoI	
TRAM 1–90	TRAM-YFP	Fwd	CATGAATTCATGGGTATCGGGAAGTCTAAA	EcoRI	
		Rev	TTACCTCGAGATGTGTCATCTTCTGCATGCAATATC	XhoI	
TRAM 1–100	TRAM-YFP	Fwd	CATGAATTCATGGGTATCGGGAAGTCTAAA	EcoRI	
		Rev	TTACCTCGAGATAGCAGATTCTGGACTCTGAGG	XhoI	
TRAM 1–120	TRAM-YFP	Fwd	CATGAATTCATGGGTATCGGGAAGTCTAAA	EcoRI	
		Rev	TTACCTCGAGACTGTCTGCCACATGGCATCTC	XhoI	
TRAM E87A/D88A/D89A	TRAM-YFP	Fwd	CATGAATTCATGGGTATCGGGAAGTCTAAA	EcoRI	
		Rev	CATCTGTGGCAGCTGCTGCATGCAATATCACAAATTTGAG		
	TRAM-YFP	Fwd	GCATGCAGCAGCTGCCACAGATGAAGCCCTCAGAGTCC		
		Rev	TTAACTCGAGCGGCAATAAATTGTCTTTGTACC	XhoI	
TRAM D91A/E92A	TRAM-YFP	Fwd	CATGAATTCATGGGTATCGGGAAGTCTAAA	EcoRI	
		Rev	CTGAGGGCTGCAGCTGTGTCATCTTCTGCATGCAA		
	TRAM-YFP	Fwd	GATGACACAGCTGCAGCCCTCAGAGTCCAGAATC		
		Rev	TTAACTCGAGCGGCAATAAATTGTCTTTGTACC	XhoI	
Other constructs					
PAK1-PBD	pDONOR-PAK	Fwd	AATTGGATCCAAGAAAGAGAAAGAGCGGCCAG		pGex-2TK (GST)
		Rev	TATAGAATTCTCAAGCTGACTTATCTGTAAAGCTCATG		
pLVX-FIP2	pEGFP-FIP2		NA	EcoRI	pLVX-EF2α- IRES-ZsGreen

### Co-immunoprecipitation

Flag-tagged proteins and EGFP-, EYFP- or ECFP-tagged proteins were overexpressed in HEK293T cells, with or without co-expression of human (h) TLR4, hCD14 and hMD2 encoding plasmids. After 48 h of transfection cells were washed with PBS and harvested in lysis buffer (150 mM NaCl, 50 mM TrisHCl pH 8.0, 1 mM EDTA, 1% NP-40) supplemented with cOmplete™, Mini, EDTA-free Protease Inhibitor Cocktail, PhosSTOP, 50 mM NaF, 2 mM NaVO3 (Sigma-Aldrich) and 2.5 U/mL Benzonase Nuclease (Novagen). Cell lysates were incubated on ice before centrifugation at 18000 x g, 4 ^o^C for 15 min, and co-immunoprecipitations performed using 30 μL of anti-FLAG M2 affinity agarose (A2220, Sigma-Aldrich) with rotation for 4 h at 4 ^o^C. After washing the immunocomplexes were eluted at 95 ^o^C for 3 min in 40 μL 2 x LDS buffer (Invitrogen). Agarose beads were removed by centrifugation at 10000 x g for 30 seconds and DTT added to 25 mM. The samples were heated for 10 min at 80 ^o^C before subjected to SDS-PAGE and immunoblotting. For endogenous co-immunoprecipitations, 5 μg rabbit anti-TICAM2/TRAM (H-85 Santa Cruz Biotechnology) antibody or normal rabbit IgG was coupled to 1.5 mg magnetic Dynabeads® M-270 Epoxy (Life Sciences Technology) and incubated with cleared lysates from human primary macrophages or THP-1 cells at 4 ^o^C for 2 h with rotation, before extensive washing in lysis buffer followed by elution by heating in 2 x LDS buffer as described.

### Rac1/Cdc42 activation assay

The partial sequence of the p21-activated kinase 1 (PAK1) (67–150 a.a) containing p21-binding domain (PBD) from pDONR223-PAK1 (a gift from William Hahn & David Root, Addgene plasmid 23543), was subcloned to pGex-2TK vector (GE Healthcare Life Sciences) using the primers 5’-AATTGGATCCAAGAAAGAGAAAGAGCGGCCAG-3’ and 5’-TATAGAATTCTCAAGCTGACTTATCTGTAAAGCTCATG-3’ with Phusion High-Fidelity DNA Polymerase (F530, Thermo Scientific) before digesting the PCR product with Fast Digest enzymes BamHI and EcoRI (Fermentas). The PAK1-PBD in pGEX-2TK was transformed to BL21 (DE3) Competent *E*. *coli* to produce GST-PAK1-PBD- recombinant protein following manufacturer’s instructions. Purified GST-PBD-PAK1 was used as a probe for pull downs of activated Cdc42 and Rac1. THP-1 cells were treated with FIP2 siRNA, TRAM siRNA or NS RNA and stimulated with *E*. *coli* bioparticles. Following stimulation, the cells were placed on ice and washed with cold PBS and lysed in lysis buffer (25 mM HEPES pH 7.2, 150 mM NaCl, 5 mM MgCl_2_, 1% Nonidet P40, 5% glycerol, 100 μM GDP). The cells were detached by scraping and the lysates cleared by centrifugation and immediately mixed with the glutathione-agarose beads conjugated with GST-PAK1-PBD and the solutions gently rotated for 45 min at 4°C. The beads were collected by centrifugation and washed two times in lysis buffer before bound proteins were eluted by heating in LDS-sample buffer (Invitrogen). GTP-bound forms of Cdc42 and Rac1 were resolved on 12% NuPage gels (Invitrogen) and detected by Western blot analysis using anti-Cdc42 antibodies (Santa Cruz Biotechnology) and anti-Rac1/2/3 (Cell Signaling Technology). The top parts of the gels were stained by Simple stain (Thermo Fisher Scientific) and GST-PBD-PAK1 imaged on Carestream GelLogic 212 PRO. The Molecular Imaging software (Carestream Health Inc) was used for quantification of GST-PBD-PAK1. Total Rac1, Cdc42, Rab11FIP2 and β-tubulin were used for the control of protein input in lysates used for the pull downs.

### Immunoblotting

Protein samples were run on pre-cast NuPAGE™ Bis-Tris gels (Invitrogen) with 1 x MES or MOPS buffer (Invitrogen) and transferred on nitrocellulose membranes, using the iBlot®2 Gel Transfer Device (Invitrogen). Membranes were washed in TBS-T (Tris Buffered Saline with 0.1% Tween-X100) and blocked with TBS-T containing 5% dry milk or 5% bovine serum albumin (BSA, Sigma-Aldrich). Membranes were incubated with primary antibodies in TBS-T containing 1% dry milk or 1% BSA at 4°C overnight or for 2–3 days. The following primary antibodies were used: anti–FLAG M2 (Sigma-Aldrich), anti-GFP (Living Colors® Full-Length GFP Polyclonal Antibody, Clontech), anti-TICAM2 (GeneTex); anti-Rab11FIP2 (ab180504), anti-β-tubulin (ab15568), anti-IRF3 (ab68481) and anti-phospho IRF3 S386 (ab192796) and from Abcam; anti-phospho IRF3 (S396) (4D4G) and (S386), anti-phospho TBK1 (S172), anti-phospho IκBα (I4D4), anti-phospho p38MAPK (T180/Y182), anti-MyD88 (D80F5), anti-Rab11 (D4F5 XP), anti-TRIF and anti-Rac1/2/3 all from Cell Signaling Technology; anti-Rab11FIP2 (S-17), anti-Cdc42 (P1) and anti-PCNA (FL-261) from Santa Cruz Biotechnology. Membranes were washed in TBS-T and incubated with secondary antibodies (HRP-conjugated, DAKO) for 1 h at room temperature in TBS-T containing 1% milk or BSA, developed with SuperSignal West Femto Substrate (Thermo Scientific) and captured with LI-COR Odyssey system. Images were analyzed by Odyssey Image Analysis software.

### Immunostaining and confocal microscopy

THP-1 cells or human PBMC derived macrophages were seeded in 24-well glass-bottom plates (MatTek Corporation) and fixed with a 1:1 solution of methanol: acetone for minimum 1h at -20°C or 2% paraformaldehyde (PFA) as previously described [10]. Immunostaining was performed after blocking in 20% human serum in PBS, using the following primary antibodies diluted to 2 μg/mL in 2% serum in PBS: rabbit anti-TICAM2/TRAM (H-85), rabbit-anti-TLR4 (H-80), goat anti-Rab11FIP2 (Santa Cruz Biotechnology). Normal rabbit IgG and goat IgG (Santa Cruz Biotechnology) were used as controls for antibody specificity. Highly cross-absorbed secondary antibodies used for confocal microscopy (Invitrogen): Goat anti-Rabbit IgG (H+L) Alexa Fluor 647 (A-21244), Goat anti-Rabbit IgG (H+L) Alexa Fluor 488 (A-11034), Donkey anti-Goat IgG (H+L) Alexa Fluor 647 (A-21447) and Donkey anti-Goat IgG (H+L) Alexa Fluor 555 (A-21432) were used at a concentration of 1 μg/mL in 2% serum in PBS. Phalloidin Alexa Fluor 488 (A-12379, Invitrogen) and Rhodamine Phalloidin (R415, Invitrogen) were used for F-actin staining. Confocal images were captured using a Leica TCS SP8 (Leica Microsystems) equipped with a HC plan-apochromat 63×/1.4 CS2 oil-immersion objective and the LAS X software, using 488 nm, 561 nm and 633 nm white laser lines and the 405 nm laser for detection. Three-dimensional data was obtained from 12-bit raw imaging data used to the build individual Z-stacks for the individual *E*. *coli* or *S*. *aureus*, F-actin-, TLR4-, TRAM- and FIP2-channels. The Bitplane-IMARIS 8.4.2 software and the inbuild spot detection mode were used to define individual phagosomes. The data were presented as median fluorescence voxel intensity of phagosomes or antibody staining on phagosomes. The software produced numerical values that were tested using the GraphPad-PRISM 6.0 and found not to show a Gaussian distribution. Therefore, statistical significance was calculated by One-way ANOVA Kruskal-Wallis multiple comparison test, reporting multiplicity adjusted p values (adj. p values). For single comparisons the Mann-Whitney test was used.

Stimulated emission depletion (STED) microscopy was used to investigate the localization of TRAM on phagosomal membranes of fixed cells using a Goat anti-rabbit IgG STAR RED secondary antibody (2-0012-011-9, Abberior) and embedded in ProLong® Diamond Antifade Mountant (P36970, Invitrogen). STED images were acquired on a Leica TCS SP8 STED-3X microscope with a 100×STED objective (HC PL APO 100×1.4 oil) and STED 775 nm depletion laser combined with the 488 and 561 nm white laser lines in the regular confocal mode for the companion markers. 3D-STED images, 16-bit raw data, were deconvolved using SVI Huygens before generating single micrographs or performing 3D-rendering using the Bitplane-IMARIS 8.4.2 software.

### Flow cytometry

A flow cytometry-based phagocytic assay was used to measure the phagocytic efficiency of pHrodo-conjugated *E*. *coli* and *S*. *aureus* BioParticles in THP-1 cells, *Tlr4*^*-*^*/*^*-*^, *Tram*^-/-^, *Myd88*^*-/-*^ iBMDMs from C57BL/6 mice [46]. According to the manufacturer, the pHrodo® dye conjugates are low-fluorescent outside the cell but fluoresce brightly in phagosomes following uptake. Prior to being added to cells the bacterial bioparticles were opsonized in 10% human A^+^ or normal mouse serum (sc-45051, Santa Cruz Biotechnology). After stimulation, cells in 6-well plates, were put on ice and washed with cold PBS before being detached by treatment with 500 μl of Accutase solution for 10–15 min (Sigma-Aldrich) and transferred into FACS tubes. The cells were washed with PBS followed by PBS containing 2% FCS. The fluorescence intensity was measured with a BD LRSII flow cytometer using the FACS Diva software (BD Biosciences). Data were exported and analysed with FlowJo software v10.0.5 (Tree Star).

### Phagocytic assay of live bacteria

Cells were seeded at a density of 200 000 cells /well in in 24-well plates and serum-free medium was added to cells before infection. Live *E*. *coli* (DH5α) and *S*. *aureus* (protein A negative subsp. aureus strain Wood 46) were grown to optical density of 0.35 at 600 nm, washed with PBS and given at a dose of 10–50 bacteria per cell in 4 to 5 biological replicates. Bacteria were centrifuged onto macrophage monolayers at 750 x g for 7 min at 4°C. Plates were warmed to 37°C in a water bath for 15 min and quickly transferred to ice where each well was washed 3 times with ice-cold PBS to remove extracellular bacteria. Warm medium with 10% FCS and 100 μg/ml gentamycin were added and cells incubated for 30 min at 37°C. Subsequently, the plates were transferred to ice and again washed 3x with cold PBS. To free phagocytosed bacteria cells were lysed in 1 ml sterile water. Viable counts were determined by plating 10 μl of 1 ml lysate (diluted 10 to 20-fold) onto LB agar plates that were incubated at 37°C overnight. Colony forming units (cfu) were counted and the number of bacteria per cell calculated. To normalize the cell number per well, the total protein concentration in cell lysates was calculated using the Pierce™ BCA Protein Assay Kit, according to manufacturer’s instructions. The data were found to follow the Gaussian distribution and statistical significance was therefore calculated by the ordinary one-way ANOVA Holm-Sidak´s multiple comparisons test reporting adj. p values.

### Nanostring

Total RNA from human macrophages from 7 healthy donors treated with NS RNA or FIP2 siRNA and stimulated with *E*. *coli* particles, were hybridized with the Nanostring HS_Immunology_v2_C2328 probe set and analysed according to the manufacturer´s protocol. Count data was collected at maximum resolution and imported to R/Bioconductor 3.4.1/3.5 [51] using the NanoStringNorm 1.1.21 package without its internal normalization functions. Probes with counts in the range of the average of all non-targeting probes plus 2 standard deviations were excluded from further analysis. The count data was voom transformed, cyclic loess normalized and analysed for differential expression in limma 3.32.5 [52]. The individual donors were used as blocking factor in the linear model. Genes showing an absolute expression log2-fold change > 1.25 and FDR < 0.05 compared to samples treated with NS RNA for each time point were considered differential expressed. Hyper geometric enrichment analysis for Gene Ontologies of Biological Processes (GO BP) was performed using clusterProfiler 3.4.4 [53]. Networks were constructed based on edges between GO BP terms sharing similar genes in the analysed gene set, node size was assigned according to the ratio of differential expressed genes in each GO term and coloured according to the absolute maximum log-fold change of genes in each GO term. The resulting network was imported to Cytoscape 3.4.0 for visualization.

### Ethics statement

Human monocytes were isolated from peripheral blood mononuclear cells (PBMCs) as previously described [10]. Approval no. 2009/224 was received from the Regional Committees for Medical and Health Research Ethics (REC Central, Møre og Romsdal, Sør-Trøndelag and Nord-Trøndelag counties) for the use of Buffy coats to isolate PMBCs. Buffy coats were isolated from anonymized blood donors at the department of Immunology and Transfusion Medicine, St Olavs Hospital.

## Supporting information

S1 Fig FIP2 selectively controls *E. coli*-stimulated induction of IFNβ mRNA, related to Fig 1. (**A**) Quantification of *E*. *coli*-stimulated IFN-β mRNA. (**B**) Quantification of *E*. *coli*-stimulated TNF mRNA. (**C**) Knock down levels in THP-1 cells silenced for FIP1, FIP2, FIP3, FIP4 or FIP5. (**D**) Levels of FIP2 and FIP5 mRNA in FIP2 silenced THP-1 cells with corresponding *E*. *coli*-or LPS-stimulated induction of IFN-β mRNA. The cells were stimulated with *E*. *coli* as indicated and GAPDH mRNA levels were used for normalization. Mean + SD of one representative out of three experiments.
(TIF)

S2 Fig FIP2 and TRAM are both involved in the control of TLR4 recruitment to developing *E. coli* phagosomes, related to Fig 2 and S1 Movie. Representative images of human primary macrophages (Mϕ) stimulated with *E*. *coli* bioparticles for 15+15 min and stained for F-actin using phalloidin (cyan), and immunostained for TLR4 (green) in cells treated with NS RNA (**A**), FIP2 siRNA (**B**) or TRAM siRNA (**C**). (**D**) Time-lapse micrographs of selected time points from Movie 1. TRAM-mCherry cells (green) engulfing live *E*. *coli* expressing pZE27GFP (red) Scale bars = 5 μm.
(TIF)

S3 Fig The FIP2 recruitment to *E. coli* phagosomes is impaired in *Tlr4*^-/-^ iBMDMs, related to Fig 2. Representative images of mouse wild type and *Tlr4*^-/-^ immortalized bone-derived-macrophages (iBMDMs) stimulated with *E*. *coli* pHrodo-conjugated bioparticles for 15 min and stained for F-actin using phalloidin (cyan), and immune-stained for FIP2. (**A**) Wild type iBMDMs. (**B**) *Tlr4*^-/-^ iBMDMs. (**C**) FIP2 levels on *E*. *coli* phagosomes in wild type and *Tlr4*^-/-^ iBMDMs stimulated for 15 and 15+15-min. One-way ANOVA Kruskal-Wallis with adj. p values, **** (p = 0.0047), (p < 0.0001). Red bars = mean ± SD. Scale bars = 5 μm. Data are representative of three independent experiments.
(TIF)

S4 Fig Rab11 binds TRAM via FIP2 and is required for optimal TRAM-FIP2 complex formation, related to Fig 4. (**A**) Immunoblot of FLAG-FIP2 pulldowns in HEK293T cells expressing FLAG-FIP2 and/or TRAM-YFP and treated with NS RNA or Rab11a- and Rab11b siRNA. (**B**) Immunoblot of FLAG-FIP2 or FLAG-FIP2 I481E pulldowns, in HEK293T cells expressing FLAG-FIP2, FLAG-FIP2 I481E or FLAG-empty vector and TRAM-YFP with or without mCherry-Rab11a. Anti-FLAG M2 agarose was used to precipitate the FLAG-FIP2 variants from lysates of HEK293T cells as indicated. (**C**) Immunoblot of FLAG-FIP2 pulldowns in HEK293T cells expressing CFP-Rab11a, CFP-Rab11aQ70L, CFP-Rab11aS25N or CFP. (**D**) HEK293 hTLR4 cells co-expressing Rab11Q70L-CFP, CD14/MD2, TRAM-YFP and FIP2-GFP stimulated for 60 min with Cy5-LPS. (**E**) HEK293 hTLR4 cells co-expressing Rab11S25N-CFP, CD14/MD2, TRAM-mCherry and FIP2-GFP stimulated for 60 min with Cy5-LPS. Arrows—enlarged LPS endosomes. Bar = 5 μM. Data are representative of three independent experiments.
(TIF)

S5 Fig TRAM and MyD88 are both involved in the regulation of *E. coli* phagocytosis downstream of TLR4, related to Fig 3. (**A**) Quantification of TRAM- and MyD88 mRNAs in human primary macrophages shown in Fig 3A–3C, silenced for TRAM or MyD88 and stimulated with *E*. *coli* bioparticles as indicated. (**B**) Quantification of TRAM- and MyD88 mRNAs in THP-1 cells silenced for TRAM or MyD88. (**C**) Immunoblot of MyD88 in THP-1 cells silenced for TRAM or MyD88. (**D**) Quantification of TLR2- versus TLR4 stimulated TNF and IL-6 mRNA induction in MyD88 silenced THP-1 cells. Pam3CSK4 (1.0μg/ml) and LPS K12 (100 ng/ml) were used for stimulations. (**E**) *E*. *coli* phagocytosis in THP-1 cells 15 min and 30 min after stimulation. (**F**) *S*. *aureus* phagocytosis in THP-1 cells 15 min and 30 min after stimulation. Phagocytosis was monitored by 3-D confocal microscopy and presented as mean bacterial count per cell. One-way ANOVA Kruskal-Wallis test with adj. P values, ** = (p < 0.0083), **** = (p < 0.0001). n = number of cells investigated. (**G**) THP-1 cells treated with NS RNA, TRAM siRNA and MyD88 siRNA and stimulated with *E*. *coli* or *S*. *aureus* bioparticles. (**H**) iBMDMs from wild type, *Tram*^-/-^ and *Myd88*^-/-^ C57BL/6 mice stimulated with *E*. *coli* or *S*. *aureus* bioparticles. (**I**) iBMDM´s from wild type and *Tlr4*^-/-^ stimulated with *E*. *coli* or *S*. *aureus* bioparticles. Phagocytosis was measured by flow cytometry after indicated times of stimulation. One representative out of three or more experiments.
(TIF)

S6 Fig Inhibition of actin polymerization and FIP2 expression have similar effects on *E. coli*- and *S. aureus* phagocytosis, related to Fig 5. (**A**) FIP2 mRNA levels in FIP2 silenced primary human macrophages stimulated with *E*. *coli* bioparticles. (**B**) FIP2 mRNA levels in FIP2 silenced THP-1 cells. (**C**) THP-1 cells treated with FIP2 siRNA or NS RNA followed by incubation with 3 μM CytoD or DMSO prior to stimulation with *E*. *coli* bioparticles for 30 min. (**D**) THP-1 cells treated with FIP2 siRNA or NS RNA followed by incubation with 3 μM CytoD or DMSO prior to stimulation with *S*. *aureus* bioparticles for 30 min. Phagocytosis was monitored by flow cytometry shown and given as mean fluorescence intensity (MFI) (C and D). (**E**) Phagocytosis of *E*. *coli* bioparticles in FIP2- or Rab11-silenced human primary macrophages (Mφ) from three human donors. (**F**) Phagocytosis of *E*. *coli* bioparticles in FIP2- or TRAM-silenced Mφ from three human donors. Phagocytosis was quantified using 3-D confocal microscopy. One-way ANOVA Kruskal-Wallis with adj. p values, ** (p < 0.0001), **** (p < 0.0001). n = number of cells monitored per condition. Red bars: mean ± SEM, n = 3 experiments (E and F). One representative out of three or more experiments in (A-D).
(TIF)

S7 Fig Rac1 and Cdc42 mRNA levels in FIP2 and TRAM silenced THP-1 cells, related to Fig 5. (**A**) Rac1, Cdc42 and FIP2 mRNA levels in FIP2 silenced THP-1 cells. Average of 3 or 4 experiments. (**B**) Rac1, Cdc42 and TRAM mRNA levels in TRAM silenced THP-1 cells. Average of 5 experiments. The respective mRNA levels in NS RNA, FIP2 siRNA and TRAM siRNA were quantified using q-PCR on RNA from unstimulated THP-1 cells. Mann-Whitney test, * (p = 0.029), ** (p = 0.0079). Bars: mean ± SEM.
(TIF)

S8 Fig FIP2 silenced THP-1 cells have reduced activation of TBK1, IκBα and IRF3 in response to *E. coli* and LPS, related to Fig 8. (**A**) Quantification of LPS- and *E*. *coli-*stimulated phospho-TBK1, IRF3, IκBα, and p38 MAPK from immunoblots. Mean± SEM from 3 independent experiments. (**B**) *E*. *coli*-stimulated IRF3 and p38 MAPK phosphorylation patterns in THP-1 cells pretreated with TBK1 inhibitors MRT67307 and BX-795. (**C**) Quantification of *E*. *coli*-stimulated IRF3 and p38 MAPK phosphorylation patterns in (B). (**D**) Effect of TBK1 inhibitors on *E*. *coli* phagocytosis in THP-1 cells. (**E)** Effect of TBK1 MRT67307 on *E*. *coli* and *S*. *aureus* phagocytosis in THP-1 cells. (**F**) Effect of TBK1 inhibitors on phagocytosis in primary human macrophages. The cells were pretreated with 1.0 μM inhibitor for 30 min prior stimulation with *E*. *coli* or *S*. *aureus* bioparticles for 15 min and phagocytosis quantified by 3-D confocal microscopy (D- F). Red bars: mean ± SD. n = number of cells monitored per condition. One-way ANOVA Kruskal-Wallis test (D-E) or Holm-Sidak´s test with adj. p values (F), ** (p < 0.0024), **** (p < 0.0001). One representative out of three independent experiments.
(TIF)

S9 Fig The effect on FIP2 silencing on *E. coli* stimulated gene expressions in human macrophages, related to Fig 8. (**A**) Effect of FIP2 silencing on *E*. *coli*-stimulated induction of IFN-β, CXCL9, CXCL10, CXCL11 and TL12B mRNA levels. (**B**) Effect of FIP2 silencing on the *E*. *coli*-stimulated induction of TNF, TLR4, CD14 and FIP2 mRNA levels. The *E*. *coli* stimulated induction of mRNA levels form the 7 human donors analyzed in Fig 8. Mann-Whitney test, * (p< 0.038), ** (p < 0.0041). Bars: mean ± SEM.
(TIF)

S1 Table Transcriptome profiling in unstimulated primary human macrophages treated with FIP2 siRNA versus NS RNA, related to Fig 8. (XLSX)

S2 Table Transcriptome profiling in unstimulated primary human macrophages treated with FIP2 siRNA versus NS RNA following 2h of *E. coli* stimulation, related to Fig 8. (XLSX)

S3 Table Transcriptome profiling in unstimulated primary human macrophages treated with FIP2 siRNA versus NS RNA following 4h of *E. coli* stimulation, related to Fig 8. (XLSX)

S1 Movie TRAM is rapidly recruited to developing *E. coli* phagosomes, related to Figs 1 and 2. THP-1 cell line expressing TRAM^mCherry^ were added live *E*. *coli* expressing pZE27GFP. The uptake of bacteria monitored for a period of 13 min and 14 s. TRAM^mCherry^ (Green) and *E*. *coli* (red).
(MP4)
