# Supplementary figures and images for: The TLR4 adaptor TRAM controls the phagocytosis of Gram-negative bacteria by interacting with the Rab11-family interacting protein 2
Source: PLoS Pathog. 2019 Mar 18;15(3):e1007684. doi: 10.1371/journal.ppat.1007684 (PMC6438586; doi:10.1371/journal.ppat.1007684)

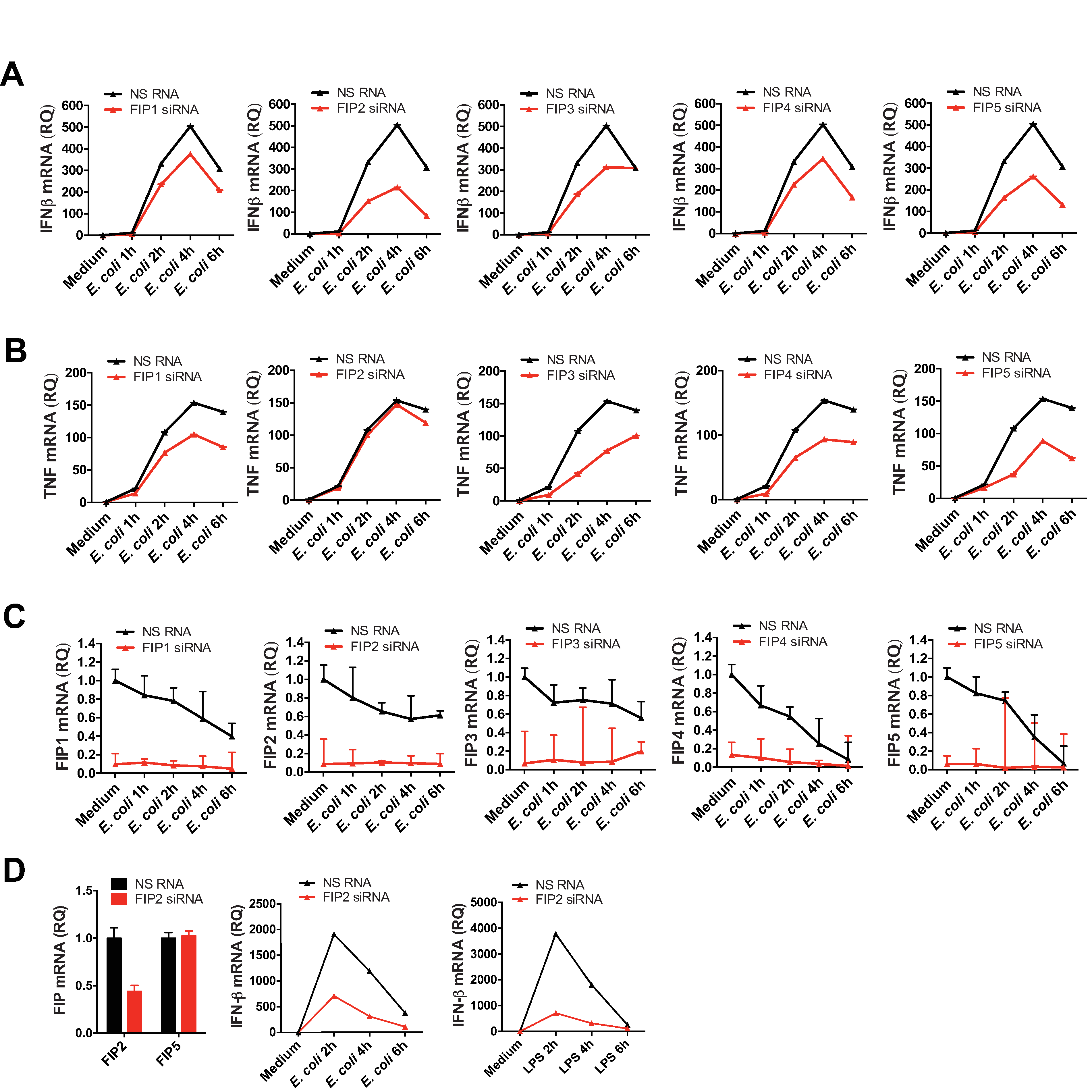

Supplement: S1 Fig — (A) Quantification of E. coli-stimulated IFN-β mRNA. (B) Quantification of E. coli-stimulated TNF mRNA. (C) Knock down levels in THP-1 cells silenced for FIP1, FIP2, FIP3, FIP4 or FIP5. (D) Levels of FIP2 and FIP5 mRNA in FIP2 silenced THP-1 cells with corresponding E. coli-or LPS-stimulated induction of IFN-β mRNA. The cells were stimulated with E. coli as indicated and GAPDH mRNA levels were used for normalization. Mean + SD of one representative out of three experiments. (TIF) [file ppat.1007684.s001.tif]

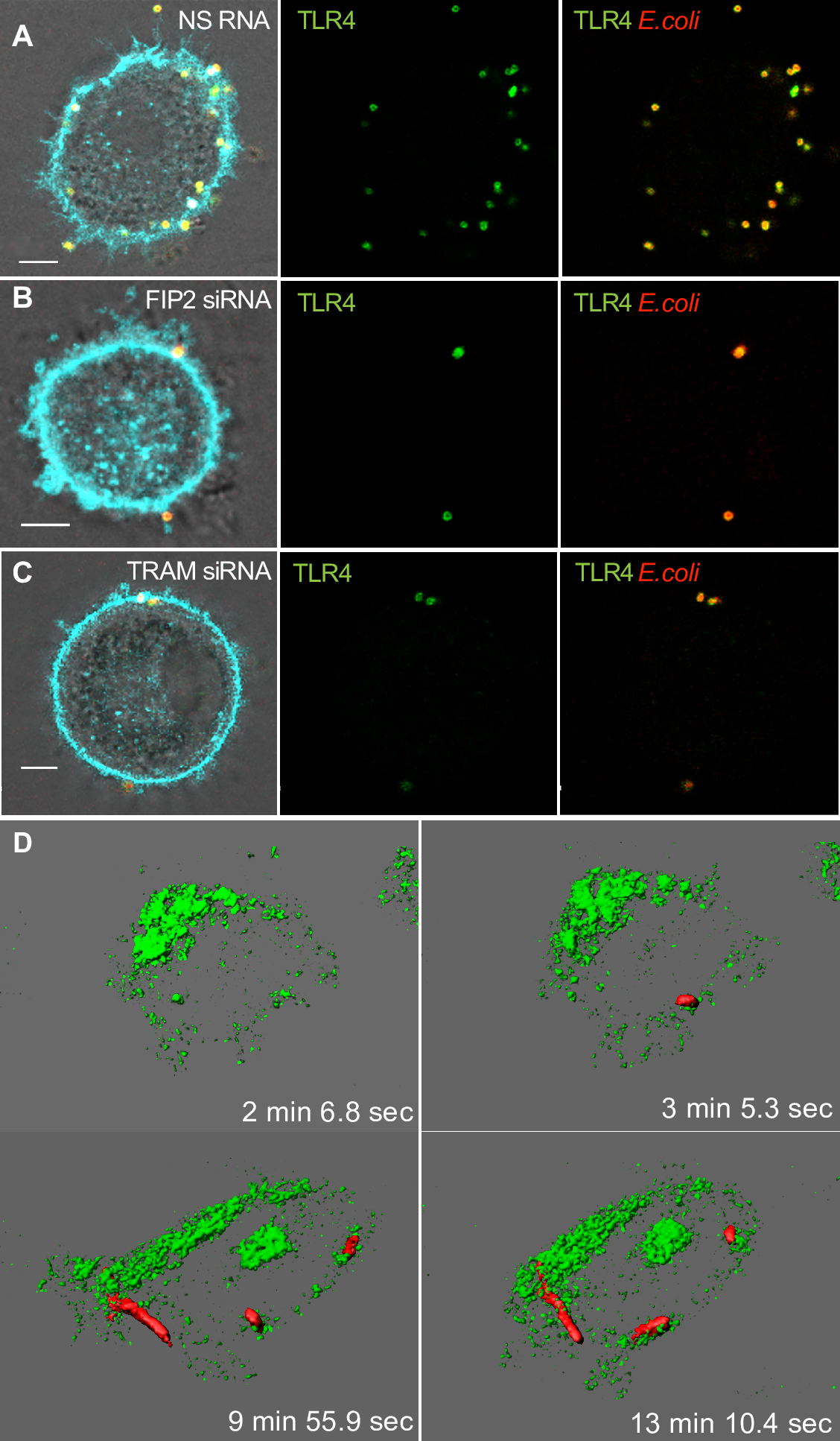

Supplement: S2 Fig — Representative images of human primary macrophages (Mϕ) stimulated with E. coli bioparticles for 15+15 min and stained for F-actin using phalloidin (cyan), and immunostained for TLR4 (green) in cells treated with NS RNA (A), FIP2 siRNA (B) or TRAM siRNA (C). (D) Time-lapse micrographs of selected time points from Movie 1. TRAM-mCherry cells (green) engulfing live E. coli expressing pZE27GFP (red) Scale bars = 5 μm. (TIF) [file ppat.1007684.s002.tif]

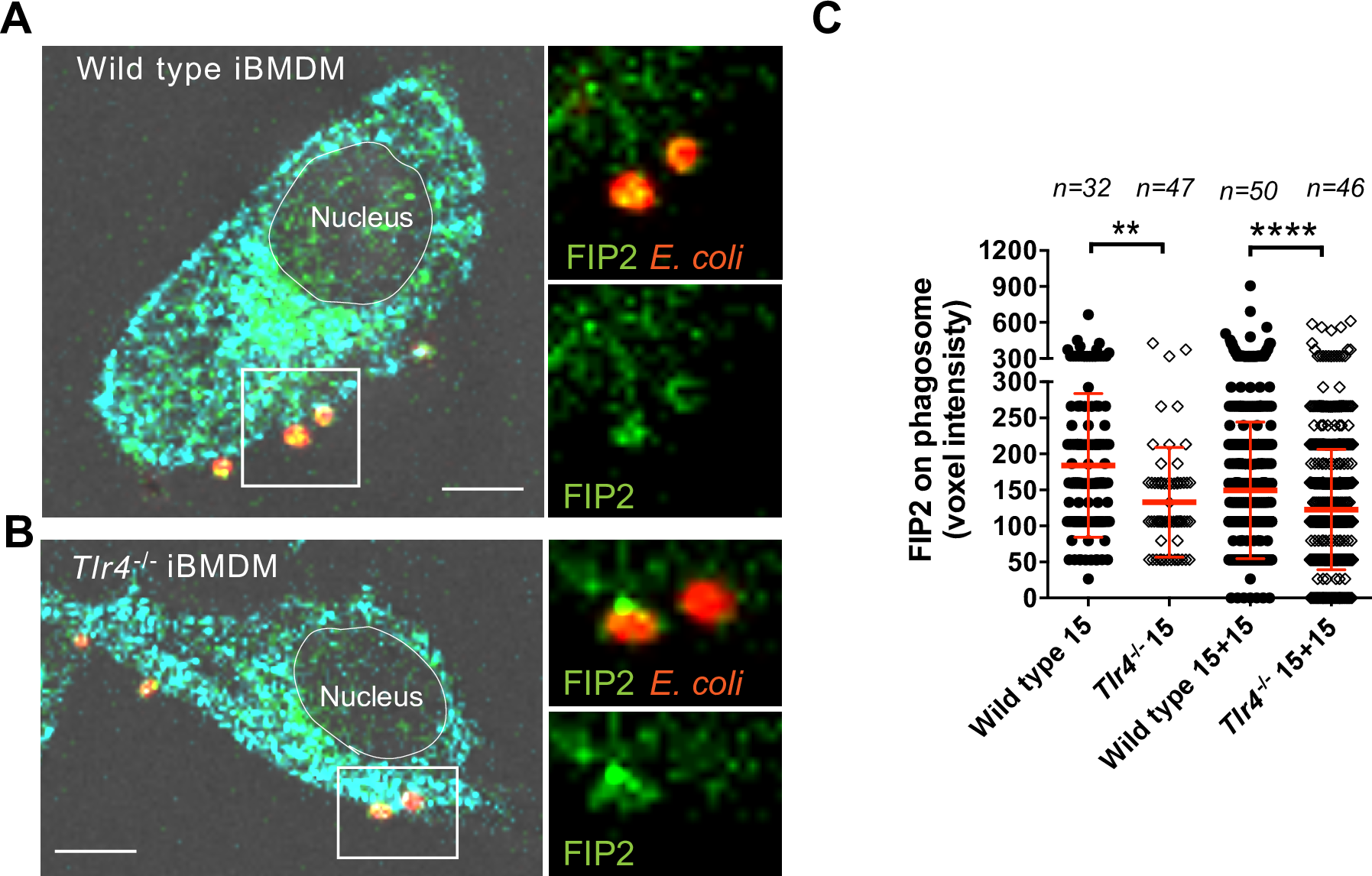

Supplement: S3 Fig — Representative images of mouse wild type and Tlr4-/- immortalized bone-derived-macrophages (iBMDMs) stimulated with E. coli pHrodo-conjugated bioparticles for 15 min and stained for F-actin using phalloidin (cyan), and immune-stained for FIP2. (A) Wild type iBMDMs. (B) Tlr4-/- iBMDMs. (C) FIP2 levels on E. coli phagosomes in wild type and Tlr4-/- iBMDMs stimulated for 15 and 15+15-min. One-way ANOVA Kruskal-Wallis with adj. p values, **** (p = 0.0047), (p < 0.0001). Red bars = mean ± SD. Scale bars = 5 μm. Data are representative of three independent experiments. (TIF) [file ppat.1007684.s003.tif]

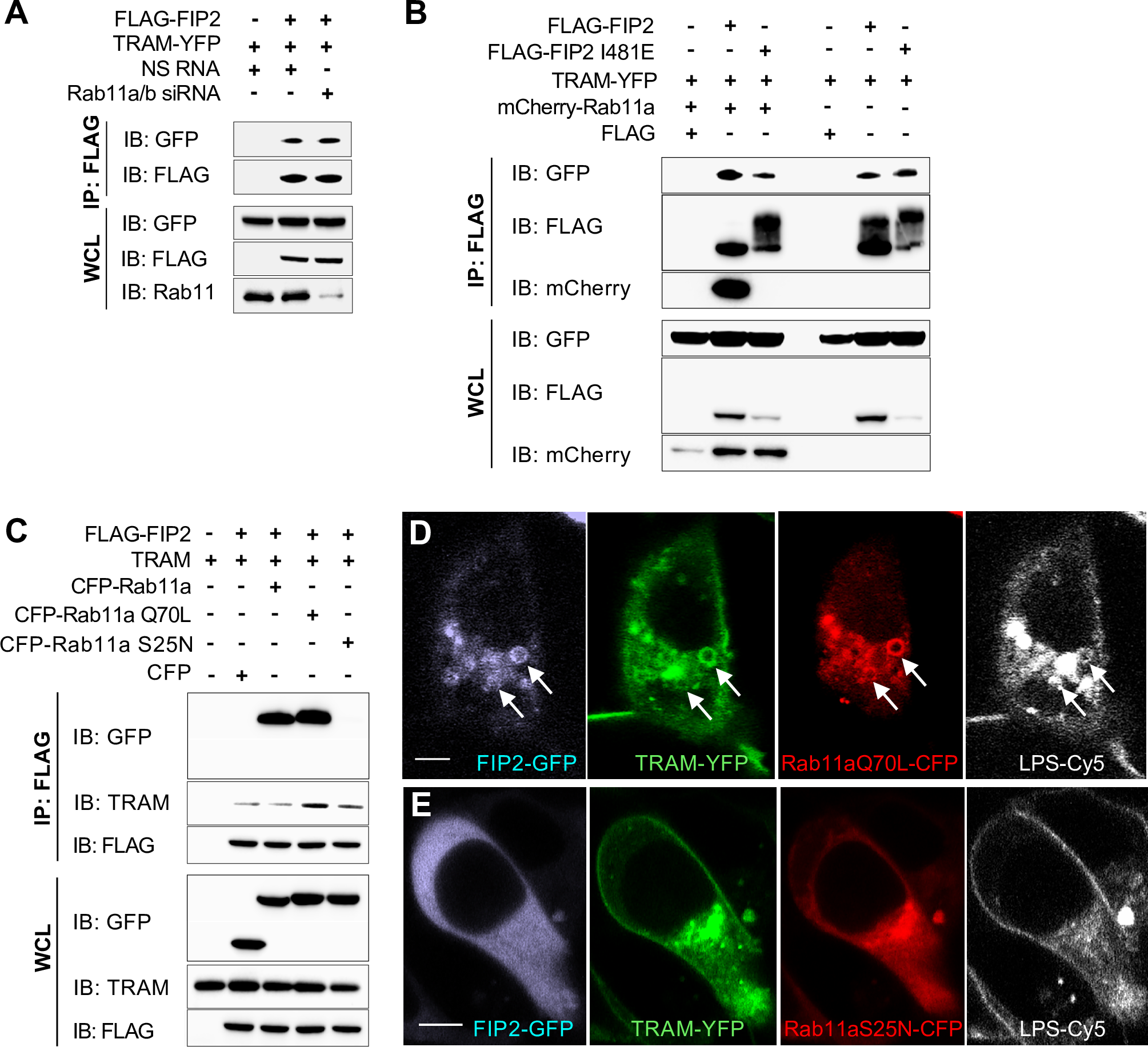

Supplement: S4 Fig — (A) Immunoblot of FLAG-FIP2 pulldowns in HEK293T cells expressing FLAG-FIP2 and/or TRAM-YFP and treated with NS RNA or Rab11a- and Rab11b siRNA. (B) Immunoblot of FLAG-FIP2 or FLAG-FIP2 I481E pulldowns, in HEK293T cells expressing FLAG-FIP2, FLAG-FIP2 I481E or FLAG-empty vector and TRAM-YFP with or without mCherry-Rab11a. Anti-FLAG M2 agarose was used to precipitate the FLAG-FIP2 variants from lysates of HEK293T cells as indicated. (C) Immunoblot of FLAG-FIP2 pulldowns in HEK293T cells expressing CFP-Rab11a, CFP-Rab11aQ70L, CFP-Rab11aS25N or CFP. (D) HEK293 hTLR4 cells co-expressing Rab11Q70L-CFP, CD14/MD2, TRAM-YFP and FIP2-GFP stimulated for 60 min with Cy5-LPS. (E) HEK293 hTLR4 cells co-expressing Rab11S25N-CFP, CD14/MD2, TRAM-mCherry and FIP2-GFP stimulated for 60 min with Cy5-LPS. Arrows—enlarged LPS endosomes. Bar = 5 μM. Data are representative of three independent experiments. (TIF) [file ppat.1007684.s004.tif]

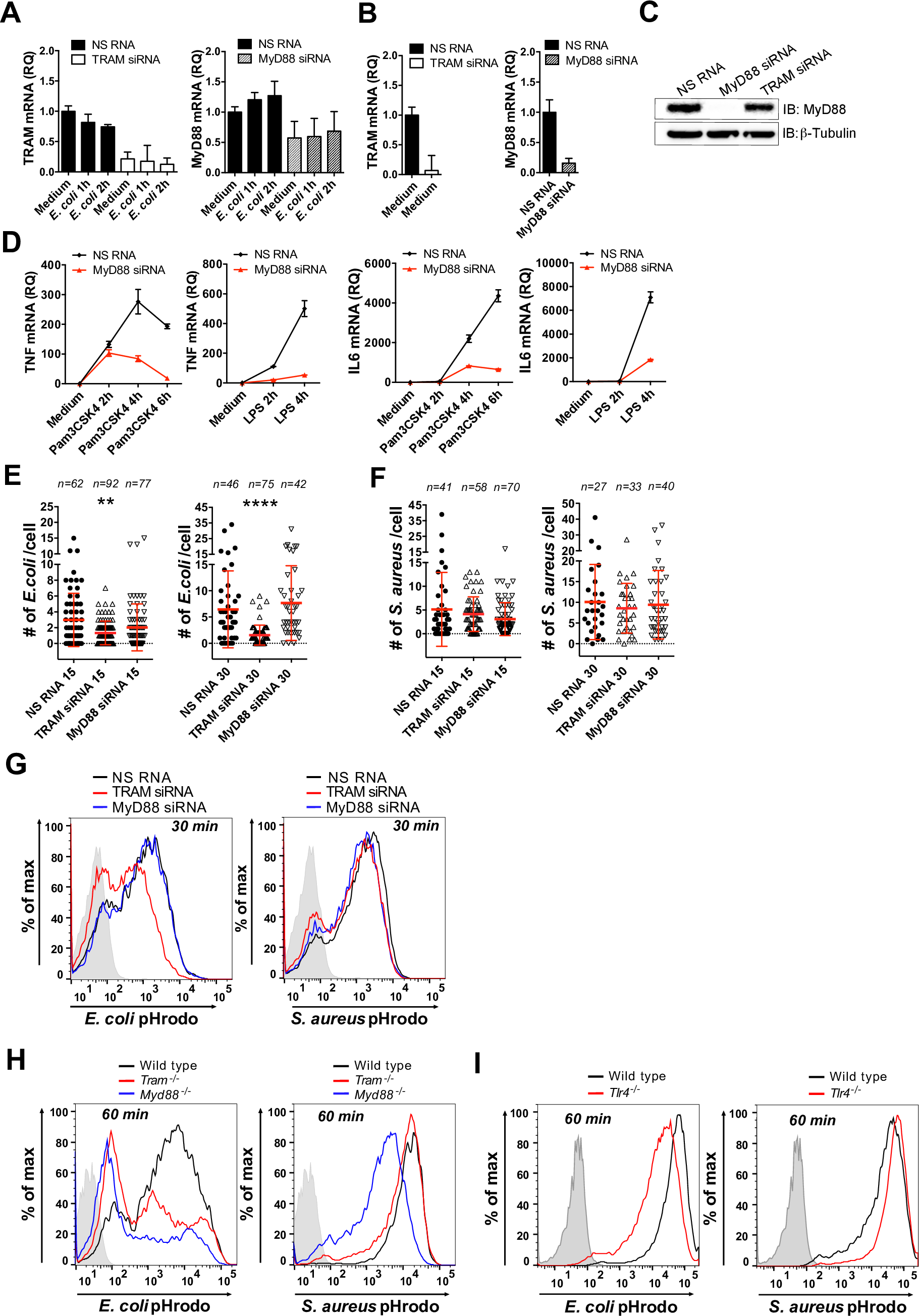

Supplement: S5 Fig — (A) Quantification of TRAM- and MyD88 mRNAs in human primary macrophages shown in Fig 3A–3C, silenced for TRAM or MyD88 and stimulated with E. coli bioparticles as indicated. (B) Quantification of TRAM- and MyD88 mRNAs in THP-1 cells silenced for TRAM or MyD88. (C) Immunoblot of MyD88 in THP-1 cells silenced for TRAM or MyD88. (D) Quantification of TLR2- versus TLR4 stimulated TNF and IL-6 mRNA induction in MyD88 silenced THP-1 cells. Pam3CSK4 (1.0μg/ml) and LPS K12 (100 ng/ml) were used for stimulations. (E) E. coli phagocytosis in THP-1 cells 15 min and 30 min after stimulation. (F) S. aureus phagocytosis in THP-1 cells 15 min and 30 min after stimulation. Phagocytosis was monitored by 3-D confocal microscopy and presented as mean bacterial count per cell. One-way ANOVA Kruskal-Wallis test with adj. P values, ** = (p < 0.0083), **** = (p < 0.0001). n = number of cells investigated. (G) THP-1 cells treated with NS RNA, TRAM siRNA and MyD88 siRNA and stimulated with E. coli or S. aureus bioparticles. (H) iBMDMs from wild type, Tram-/- and Myd88-/- C57BL/6 mice stimulated with E. coli or S. aureus bioparticles. (I) iBMDM´s from wild type and Tlr4-/- stimulated with E. coli or S. aureus bioparticles. Phagocytosis was measured by flow cytometry after indicated times of stimulation. One representative out of three or more experiments. (TIF) [file ppat.1007684.s005.tif]

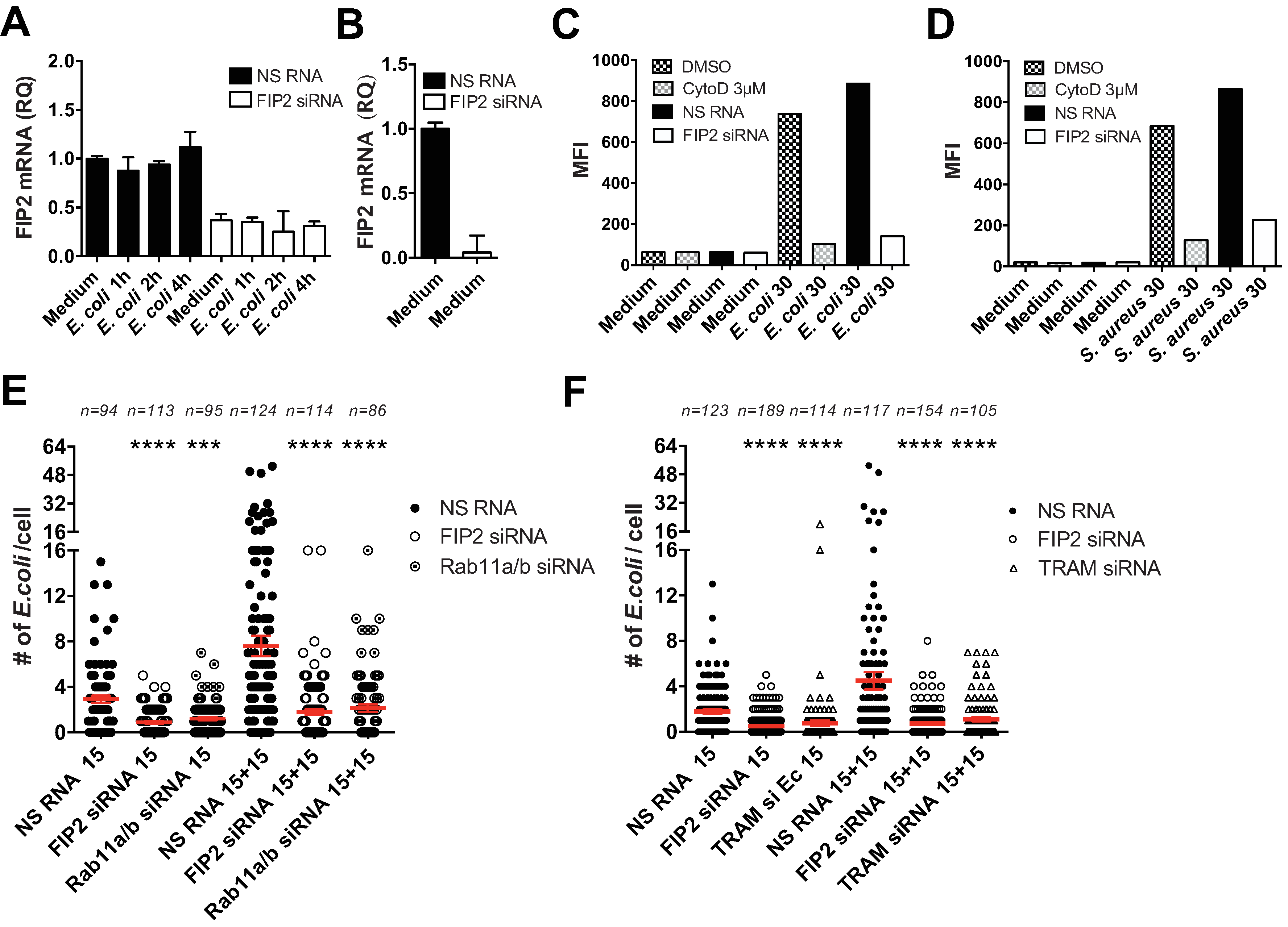

Supplement: S6 Fig — (A) FIP2 mRNA levels in FIP2 silenced primary human macrophages stimulated with E. coli bioparticles. (B) FIP2 mRNA levels in FIP2 silenced THP-1 cells. (C) THP-1 cells treated with FIP2 siRNA or NS RNA followed by incubation with 3 μM CytoD or DMSO prior to stimulation with E. coli bioparticles for 30 min. (D) THP-1 cells treated with FIP2 siRNA or NS RNA followed by incubation with 3 μM CytoD or DMSO prior to stimulation with S. aureus bioparticles for 30 min. Phagocytosis was monitored by flow cytometry shown and given as mean fluorescence intensity (MFI) (C and D). (E) Phagocytosis of E. coli bioparticles in FIP2- or Rab11-silenced human primary macrophages (Mφ) from three human donors. (F) Phagocytosis of E. coli bioparticles in FIP2- or TRAM-silenced Mφ from three human donors. Phagocytosis was quantified using 3-D confocal microscopy. One-way ANOVA Kruskal-Wallis with adj. p values, ** (p < 0.0001), **** (p < 0.0001). n = number of cells monitored per condition. Red bars: mean ± SEM, n = 3 experiments (E and F). One representative out of three or more experiments in (A-D). (TIF) [file ppat.1007684.s006.tif]

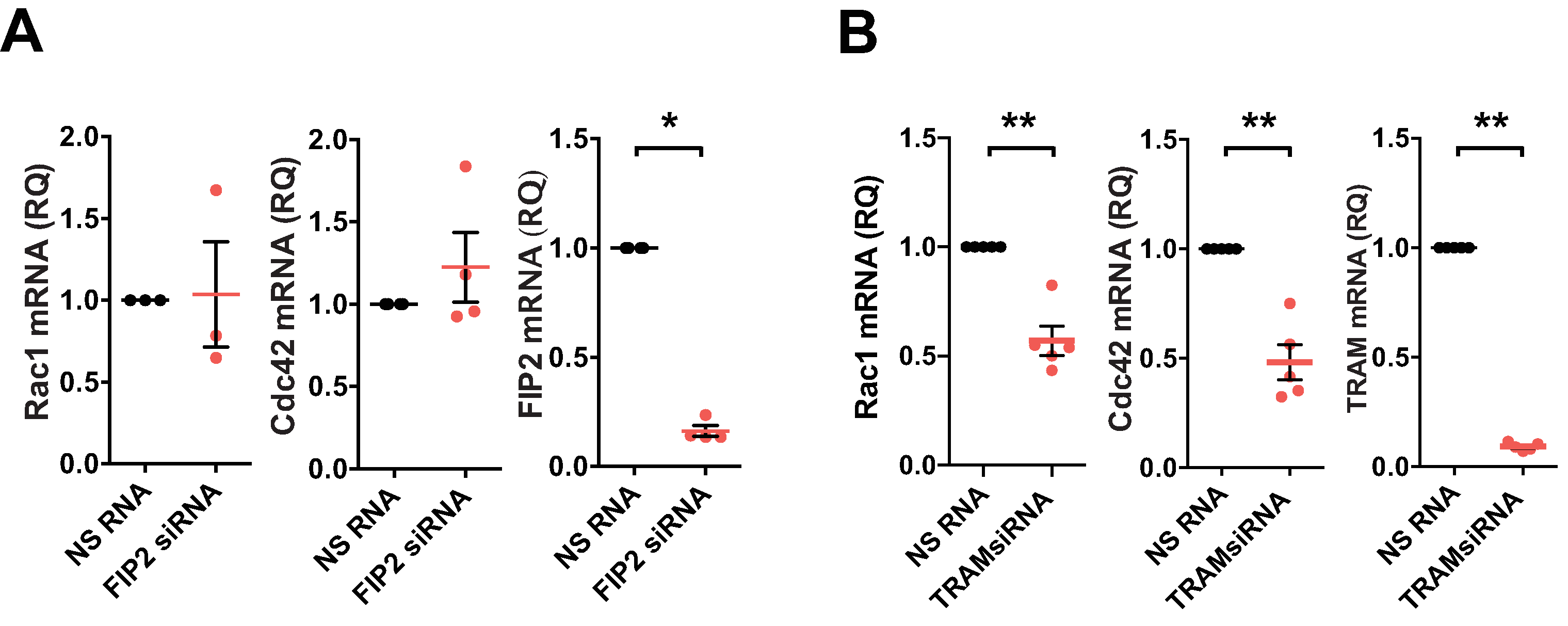

Supplement: S7 Fig — (A) Rac1, Cdc42 and FIP2 mRNA levels in FIP2 silenced THP-1 cells. Average of 3 or 4 experiments. (B) Rac1, Cdc42 and TRAM mRNA levels in TRAM silenced THP-1 cells. Average of 5 experiments. The respective mRNA levels in NS RNA, FIP2 siRNA and TRAM siRNA were quantified using q-PCR on RNA from unstimulated THP-1 cells. Mann-Whitney test, * (p = 0.029), ** (p = 0.0079). Bars: mean ± SEM. (TIF) [file ppat.1007684.s007.tif]

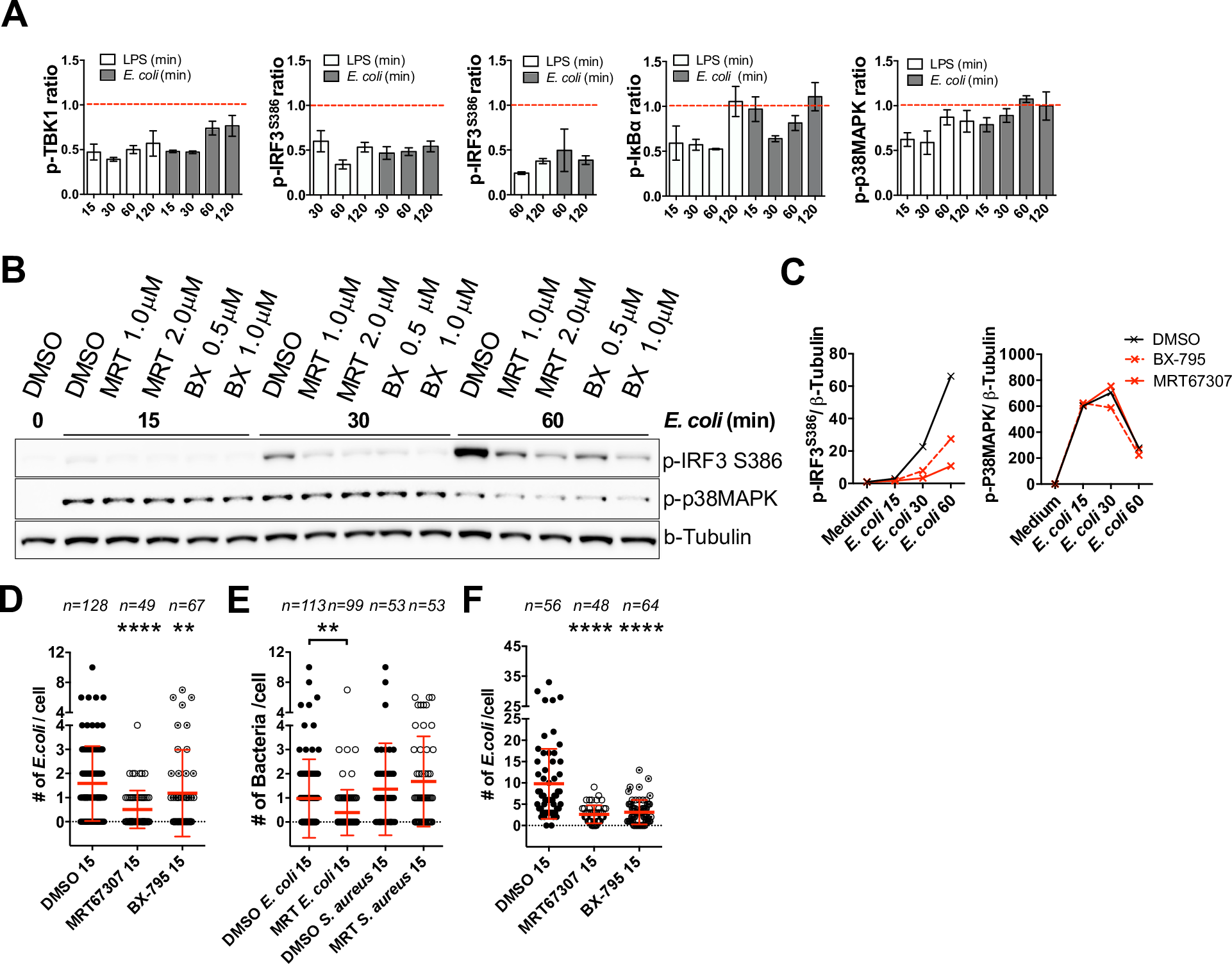

Supplement: S8 Fig — (A) Quantification of LPS- and E. coli-stimulated phospho-TBK1, IRF3, IκBα, and p38 MAPK from immunoblots. Mean± SEM from 3 independent experiments. (B) E. coli-stimulated IRF3 and p38 MAPK phosphorylation patterns in THP-1 cells pretreated with TBK1 inhibitors MRT67307 and BX-795. (C) Quantification of E. coli-stimulated IRF3 and p38 MAPK phosphorylation patterns in (B). (D) Effect of TBK1 inhibitors on E. coli phagocytosis in THP-1 cells. (E) Effect of TBK1 MRT67307 on E. coli and S. aureus phagocytosis in THP-1 cells. (F) Effect of TBK1 inhibitors on phagocytosis in primary human macrophages. The cells were pretreated with 1.0 μM inhibitor for 30 min prior stimulation with E. coli or S. aureus bioparticles for 15 min and phagocytosis quantified by 3-D confocal microscopy (D- F). Red bars: mean ± SD. n = number of cells monitored per condition. One-way ANOVA Kruskal-Wallis test (D-E) or Holm-Sidak´s test with adj. p values (F), ** (p < 0.0024), **** (p < 0.0001). One representative out of three independent experiments. (TIF) [file ppat.1007684.s008.tif]

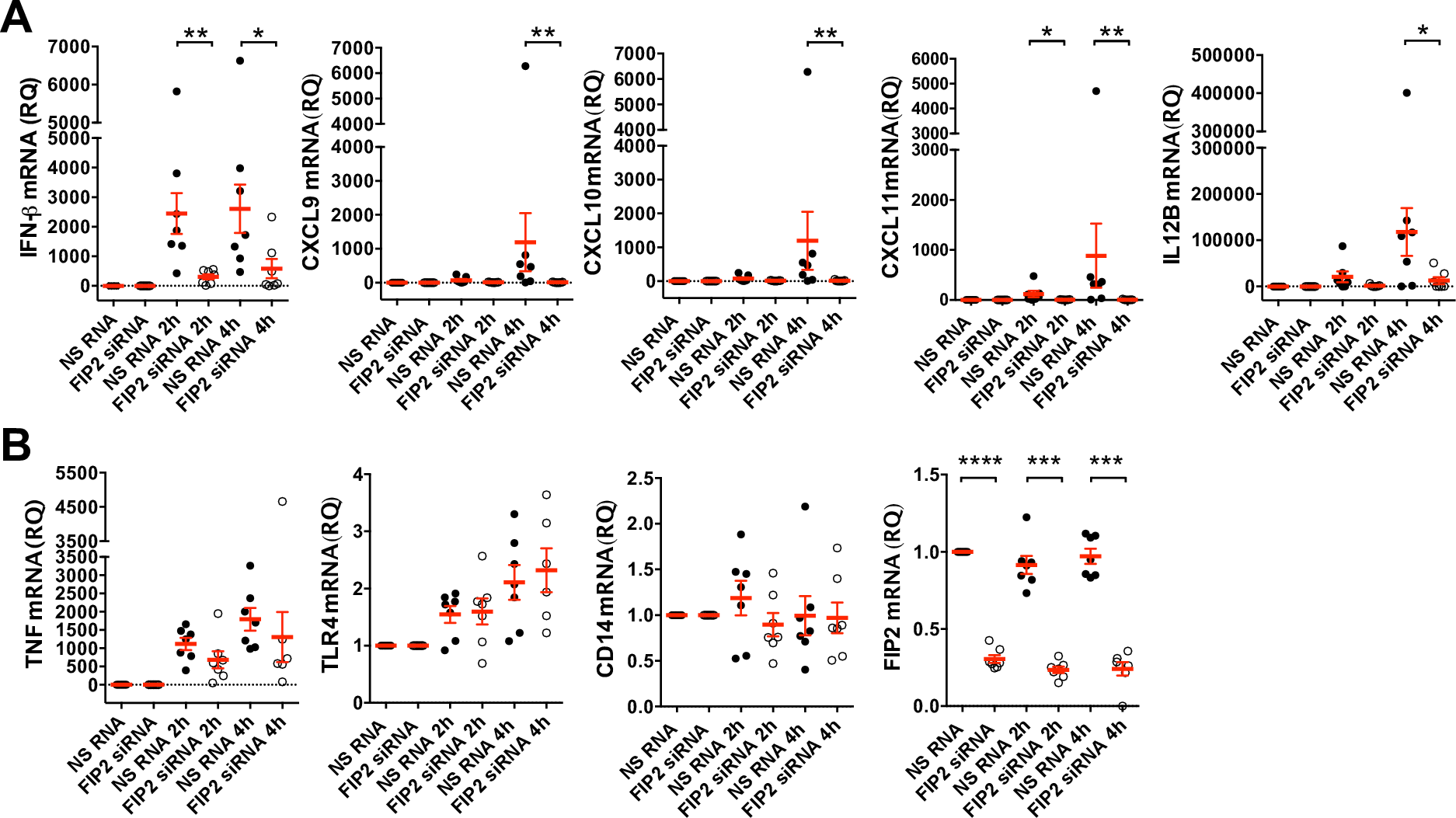

Supplement: S9 Fig — (A) Effect of FIP2 silencing on E. coli-stimulated induction of IFN-β, CXCL9, CXCL10, CXCL11 and TL12B mRNA levels. (B) Effect of FIP2 silencing on the E. coli-stimulated induction of TNF, TLR4, CD14 and FIP2 mRNA levels. The E. coli stimulated induction of mRNA levels form the 7 human donors analyzed in Fig 8. Mann-Whitney test, * (p< 0.038), ** (p < 0.0041). Bars: mean ± SEM. (TIF) [file ppat.1007684.s009.tif]
